# Inhibition of autophagy-related protein 7 enhances anti-tumor immune response and improves efficacy of immune checkpoint blockade in microsatellite instability colorectal cancer

**DOI:** 10.1186/s13046-024-03023-w

**Published:** 2024-04-16

**Authors:** Wenxin Zhang, Lu Chen, Jiafeng Liu, Bicui Chen, Huanying Shi, Haifei Chen, Huijie Qi, Zimei Wu, Xiang Mao, Xinhai Wang, Yuxin Huang, Jiyifan Li, Zheng Yu, Mingkang Zhong, Tianxiao Wang, Qunyi Li

**Affiliations:** 1 Department of Pharmacy, Huashan Hospital, Fudan University, No.12 Urumqi Middle Road, Shanghai, 200040 China; 2 Department of Surgery, Huashan Hospital, Fudan University, Shanghai, 200040 China

**Keywords:** ATG7, CD8, Cholesterol, PD-1, Colorectal cancer

## Abstract

**Background:**

The efficacy of anti-PD-1 therapy is primarily hindered by the limited T-cell immune response rate and immune evasion capacity of tumor cells. Autophagy-related protein 7 (ATG7) plays an important role in autophagy and it has been linked to cancer. However, the role of ATG7 in the effect of immune checkpoint blockade (ICB) treatment on high microsatellite instability (MSI-H)/mismatch repair deficiency (dMMR) CRC is still poorly understood.

**Methods:**

In this study, patients from the cancer genome altas (TCGA) COAD/READ cohorts were used to investigate the biological mechanism driving ATG7 development. Several assays were conducted including the colony formation, cell viability, qRT-PCR, western blot, immunofluorescence, flow cytometry, ELISA, immunohistochemistry staining and in vivo tumorigenicity tests.

**Results:**

We found that ATG7 plays a crucial role in MSI-H CRC. Its knockdown decreased tumor growth and caused an infiltration of CD8^+^ T effector cells in vivo. ATG7 inhibition restored surface major histocompatibility complex I (MHC-I) levels, causing improved antigen presentation and anti-tumor T cell response by activating reactive oxygen species (ROS)/NF-κB pathway. Meanwhile, ATG7 inhibition also suppressed cholesterol accumulation and augmentation of anti-tumor immune responses. Combining ATG7 inhibition and statins improved the therapeutic benefit of anti-PD-1 in MSI-H CRC. Importantly, CRC patients with high expression of both ATG7 and recombinant 3-hydroxy-3-methylglutaryl coenzyme A reductase (HMGCR) experienced worse prognosis compared to those with low ATG7 and HMGCR expression.

**Conclusions:**

Inhibition of ATG7 leads to upregulation of MHC-I expression, augments immune response and suppresses cholesterol accumulation. These findings demonstrate that ATG7 inhibition has therapeutic potential and application of statins can increase the sensitivity to immune checkpoint inhibitors.

**Supplementary Information:**

The online version contains supplementary material available at 10.1186/s13046-024-03023-w.

## Background

Colorectal cancer (CRC) is a global leading cause of cancer-related mortalities [1, 2]. Whilst acknowledging the significant strides in early cancer detection, most CRC cases are still diagnosed at advanced stages. Advanced metastatic colorectal cancer has a 5-year survival rate of approximately 10% after diagnosis, underscoring the urgent need for new treatment options to improve patient survival [3]. Immunotherapy has been successfully used in immune checkpoint therapy for various hematological and solid metastatic malignancies. By suppressing the interaction between T cell inhibitory receptors and their corresponding ligands, immunotherapy activates anti-tumor immune responses, causes durable tumor regression, providing novel targeted therapeutic drugs for tumors. Programmed cell death protein 1 (PD-1) and its ligand PD-L1 have displayed remarkable efficacy in patients with non-small-cell lung cancer, advanced melanoma, bladder cancer, or metastatic renal-cell carcinoma [4–7]. Pembrolizumab and nivolumab are antibodies that target programmed cell death protein 1 (PD-1). They are approved as monotherapy or combined with ipilimumab, an anti-cytotoxic T-lymphocyte-associated protein 4 antibody for treating CRC with high microsatellite instability (MSI-H)/mismatch repair deficiency (dMMR) [8]. Additional studies have demonstrated a response rate of 50% and a disease control rate of 89% among 28 patients with MSI-H tumors. However, achieving favorable outcomes for colorectal cancer patients responding to PD-1/PD-L1 therapy remains challenging, thereby necessitating more efforts to improve its efficacy [7].

Autophagy is a catabolic pathway that primarily relies on lysosomal removal of damaged or senescent organelles, holding significant implications in tumor development and anti-tumor therapy. The fusion between autophagosomes and lysosomes causes the degradation of isolated materials [9]. Studies have shown that suppressing autophagy can improve anti-tumor effects of chemotherapy or targeted therapy [10]. Our previous work also revealed important roles of autophagy in CRC treatment [11]. Furthermore, activating autophagy in tumor cells inhibits anti-tumor immune responses, providing evidence for targeting autophagy to inhibit tumor growth and improve cytotoxic T lymphocyte (CTL) and natural killer (NK)-mediated killing [12, 13]. Targeting Beclin-1, an autophagy-related gene, causes functional NK cell infiltration into the tumor microenvironment (TME) by releasing C-C motif chemokine ligand 5 (CCL5)/RANTES from tumor cells [14]. These findings suggest that activating autophagy in tumor cells is a primary mechanism for anti-tumor immune response modulation whereas improving its inhibition presents a novel approach within the field of tumor immunotherapy. Autophagy-related protein 7 (ATG7) is an important member of ATG predominantly involved in autophagosome formation. Several animal models have been adopted to explore the role of ATG7 in regulating cellular physiology by targeting autophagic processes [15].

Lipid metabolic reprogramming promotes tumor growth causing metabolic stress on tumor-infiltrating immune and stromal cells, causing impaired anti-tumor immune responses [16, 17]. Nonetheless, targeted reprogramming of lipid metabolism can suppress tumor cell growth and relieve immunosuppressive TME [18]. Previous research indicates that modifications in lipid metabolism, specifically cholesterol metabolism influence the efficacy of immunotherapy [19, 20]. In recent years, the importance of ATG7 in lipid metabolism has been increasingly acknowledged. Zhang et al. discovered that ATG7 suppression inhibits lipid metabolism [21]. Similarly, another investigation revealed that m^6^A RNA methylation regulates autophagy and lipogenesis by targeting autophagy-related protein 5 (ATG5) and ATG7 [22]. These studies suggest that ATG7 potentially influences lipid metabolism by regulating autophagy, and consequently immunotherapy.

In the present study, we used well-established immunocompetent mouse models of colorectal cancer to investigate the roles of ATG7 in immune response. The results suggested that focusing on the ATG7 gene or mitigating its kinase activity through discerning inhibitors could significantly influence the immunological milieu of colorectal cancer. This approach could potentially orchestrate the influx of CD8^+^ T effector cells into the tumor microenvironment, thereby improving its immunogenicity. We found that suppressing ATG7 increases MHC-I expression levels on the surface of cell membranes. Furthermore, we identified that ATG7 is a positive regulator of HMGCR, a target of statins that promote cholesterol accumulation in colorectal cancer. Our findings establish a novel mechanism in which ATG7 regulates cholesterol metabolism and crosstalk between MHC-I and ROS/ NF-кB, which together mediate CRC immunotherapy and provide a potential biomarker for clinically predicting the therapeutic effect of PD-1/PD-L1 blockade.

## Methods

### Cell culture

The human colorectal cancer cell lines SW480, HT-29, HCT-116, LoVo, and mouse colorectal cancer cell line MC38 were obtained from the Cell Bank of the Chinese Academy of Sciences (Shanghai, China). Cells were cultured in F12K, DMEM, McCoy’s 5 A, and RPMI 1640 (GIBCO-BRL, Carlsbad, CA, USA) containing 10% FBS (SH30084.03HI, Hyclone) at 37 °C in an atmosphere containing 5% CO_2_.

### Reagents and antibodies

ATG7 inhibitor (ATG7-IN-1) and atorvastatin were purchased from Targetmol (Boston, MA, USA). These primary antibodies included ATG7 (10088-2-AP, Proteintech), MHC-I (sc-32,235, Santa Cruz Biotechnology), HLA-A (sc-390,473, Santa Cruz Biotechnology), HLA-B (PA5-35345, Thermofisher scientific), HLA-C(ab126722, Abcam), LC3 (14600-1-AP, Proteintech), IκBα (10268-1-AP, Proteintech), H3 (17168-1-AP, Proteintech), HMGCR (sc-271,595, Santa Cruz Biotechnology), p65 (T55034F, Abmart), p-p65 (TP56372F, Abmart), GAPDH (60004-1-lg, Proteintech). Horseradish peroxidase (HRP)-conjugated secondary antibodies were obtained from Cell Signaling Technology (Danvers, MA).

### Tumor immune phenotyping and flow cytometry analysis

Tumors were harvested and mechanically dissociated into fragments within 2 h. Subsequently, the tumor tissue was disrupted to prepare single cells using a tumor isolation kit (Miltenyi Biotec) following the manufacturer’s instructions. The cell suspension was lysed with a 70 μm cell filter to remove red blood cells. Tumor-infiltrating leukocytes were isolated through gradient centrifugation using a 40%/80% Percoll (GE Healthcare) solution. Subsequently, the collected cells were blocked with Fc block (anti-mouse CD16/32, BioLegend) on ice for 30 min. The samples were first stained for surface markers for lymphoid immune populations before intracellular staining. For FoxP3 and intracellular staining, True-Nuclear Transcription Factor Buffer Set 424,401 (BioLegend) was used following the manufacturer’s instructions. The following antibodies and stain kit were purchased from BioLegend: APC-Cy7 anti-mouse CD45, Alexa Fluor 700 Hamster anti-mouse CD3e, FITC anti-mouse CD4, PerCP-Cy5.5 anti-mouse CD8a, BV786 anti-mouse CD45R/B220, PE anti-mouse NK-1.1, BV650 anti-mouse IFN-γ, Alexa Fluor 647 anti-mouse Foxp3 and fixable viability stain 510. PE-CYN7 anti-mouse granzyme B was obtained from Thermo Fisher Scientific.

### Quantitative RT-PCR (qRT-PCR)

Total RNA was isolated from the cells using the Trizol reagent (Invitrogen, Carlsbad, CA, USA). The RNA concentration was quantified via UV absorption at 260 and 280 nm. Reverse transcription was conducted using a Hifair® ® 1st Strand cDNA Synthesis SuperMix kit (Yeasen, Shanghai, China). Quantitative real-time reverse transcription polymerase chain reaction (RT-PCR) was carried out on an ABI 7500 Real-Time PCR system (Applied Biosystems, Foster City, CA, USA) using SYBR Green PCR master mix reagents (Takara, Dalian, Japan). The primers for genes were synthesized by Invitrogen (Shanghai, China). The 2^−ΔΔCt^ method was used to compute the relative quantification of the target gene mRNA level after normalization to GAPDH mRNA. Table S1 shows the primer sequences.

### Immunofluorescence

The cultured cells were washed with PBS before fixing with 4% paraformaldehyde at room temperature for 15 min. After three washes, the cells were permeabilized using 0.1% Triton X-100 in PBS at room temperature for 15 min. Subsequently, the cells were incubated with a solution containing 5% bovine serum albumin (BSA) at room temperature for one hour, followed by overnight incubation with primary antibodies at 4 °C. After washing three times with PBS, cells were incubated in secondary antibody at room temperature for 1 h. DAPI was used to label the nucleus.

### Transfections

ATG7 knockdown was investigated using the shRNA (human shATG7 or mouse shATG7) lentiviral expression system provided by Hanbio Technology (Shanghai, China). The shRNA lentiviral vectors included shATG7 (human): 5’- CAACATCCCTGGTTACAAG-3’; shATG7 (mouse): 5’-TTCTGTCACGGTTCGATAATG-3’. The negative controls were obtained from the same source. Colorectal cancer cells (6 × 10^5^) were seeded in 6-well plates and infected with ATG7 or negative control lentiviral vectors at a multiplicity of infection of 35 plaque-forming units per cell, followed by the addition of a virus that improves infection solution in a 10-fold volume. After incubation for 8 h, the medium was replaced with fresh medium containing 10% FBS and 1% antibiotics-antifungal drugs. Cell lines that stably knocked down ATG7 were selected using puromycin (Invitrogen, A1113802) at a concentration of 1 µg/mL. Trypsin digestion was performed after incubation for 72 h, and subsequent experiments were analyzed.

### Western blot analysis and ELISA

Cell and tumor tissue samples were lysed in radioimmunoprecipitation assay lysis buffer (Beyotime Biotechnology, Shanghai, China) supplemented with phosphatase and protease inhibitors. Subsequently, the lysate was centrifuged at 12,000 g at 4 °C for 10 min. The protein concentration in the resulting supernatant was quantified using Nano-400 A (Allsheng, Hangzhou, China). Equal amounts of proteins were then separated through sodium dodecyl sulfate-polyacrylamide gel electrophoresis and transferred to polyvinylidene difluoride membranes. The membranes were then blocked with 5% skim milk for 2 h and overnight incubated at 4 °C with the desired primary antibodies. This was followed by incubation with secondary antibodies (Cell Signaling Technology, USA). The visualization of proteins was achieved using an enhanced chemiluminescence system (Tiangen, Beijing, China). Chemokine concentrations of TNF-α and IFN-γ were measured using ELISA kits (Invitrogen) based on the manufacturer’s instructions.

### Measurement of serum cholesterol, TG, ALT, and AST

The levels of serum cholesterol, triglyceride (TG), alanine aminotransferase (ALT), and aspartate aminotransferase (AST) were calculated by detection kits (Nanjing Jiancheng Bioengineering Institute, Nanjing, China) following the manufacturer’s instructions.

### Cell viability and colony formation

For CCK-8 assays, 5 × 10^3^ cells/well were seeded into 96-well plates. The cell viability was assessed using the cell counting kit-8 (CCK-8) at 0, 24-, 48-, 72- and 96-hours post-seeding. Cells were incubated with CCK-8 for one hour and the absorbance at a wavelength of 450 nm was measured to determine cell viability per well. For colony formation assay, cells (1000 cells/well) were seeded in six-well plates and cultured for 10 to 14 days. Cell colonies were fixed with a solution containing 4% paraformaldehyde and stained with a solution of crystal violet at a concentration of 0.1%. Thereafter, visible colonies were photographed and manually counted.

### Measurement of ROS and GSH/GSSG ratio

For the measurement of intracellular ROS levels, 1 × 10^6^ cells were collected and incubated with 10 μm DCFH-DA (Beyotime, Shanghai, China) at 37 ℃ for 20 min. Cells were washed three times with PBS and then immediately analyzed by immunofluorescence. The reduced glutathione (GSH)/oxidized glutathione disulfide (GSSG) ratio was detected using a GSH and GSSG Assay Kit (Beyotime, Shanghai, China) based on the manufacturer’s instructions.

### NF-κB p65 translocation analyses

Cells were pretreated with ATG7 inhibition (shATG7, ATG7-IN-1 10 μm) for 24 h. Proteins from the nuclear and cytoplasm fractions were prepared using a kit from Beyotime (Shanghai, China) following the manufacturer’s instructions, and then subjected to Western blot for p65 detection. p65 translocation was verified using immunofluorescence staining with an anti-p65 antibody and PE-conjugated secondary antibody. The cells were counterstained with DAPI and observed under a Nikon fluorescence microscope (Nikon, Tokyo, Japan).

### Patient samples

The present study was approved by the Ethics Committee of Huashan Hospital under the approval number KY2021-462. A total of 91 colorectal cancer patients who consented provided their tumor tissues and para-cancerous tissues for research between May 2017 and August 2021.

### In vivo treatments

All procedures were performed based on the approved protocols by the Fudan University Institutional Animal Care and Use Committee. Animal studies were reported in compliance with the ARRIVE guidelines [23]. Male C57BL/6 mice were purchased from Shanghai Model Organisms Center, Inc (Shanghai, China). The animals were housed in a controlled environment at 23 ± 2°C under a 12 h dark/light cycle with free access to irradiated food and sterile water. Six-week-old BALB/c nude mice were subcutaneously injected with 1 × 10^7^ CRC cells in the right flank region to establish immunodeficient mouse models. C57BL/6 mice were subcutaneously injected with 1 × 10^7^ CRC cells to establish immunocompetent mouse models. After implantation, tumor growth was monitored once per 5 days. When the tumor volumes reached 100 mm^3^, mice were randomized into two groups treated with ATG7-IN-1 or vehicle control, respectively. ATG7-IN-1 was dissolved in 1% DMSO and ddH_2_O was administered to the mice at 10 mg/kg via oral gavage. For anti-PD-1 treatments, mice were intraperitoneally injected with anti-mouse anti-PD1 antibody (100 µg per mouse) or IgG isotype control (BioXCell) every 3 days for 2 weeks starting on day 7 after tumor injection. For the depletion of CD8^+^ T cells, each mouse was treated with 100 µg of anti-CD8 antibody (BioXCell) or IgG isotype control via intraperitoneal injection before tumor inoculation. Tumor growth was monitored once per 3-days after implantation. Mice were euthanized after three weeks. Tumor volumes were determined by measuring length (*l*) and width (*w*) and computing volume (*V* = 0.5 × *l* × *w*^2^) at the indicated time points. Tumor tissues were harvested for weight measurement.

### Histopathology and immunohistochemistry analyses

Tissue samples were fixed in 4% paraformaldehyde and embedded in paraffin. Tissue Sections. (5 μm) were hydrated and subjected to hematoxylin and eosin (H&E) and immunohistochemical staining. Briefly, heat-induced antigen retrieval was performed using 10 mM sodium citrate buffer, pH 6.5. Peroxidase activity was quenched with 3% H_2_O_2_ and tissues were blocked in 5% bovine serum albumin for 30 min. Primary antibody was added before incubating the sections overnight at 4 °C. Horseradish peroxidase (HRP) conjugated secondary antibody (1:1000) and DAB was used for detection. Sections were counterstained with hematoxylin. Quantification of IHC images was performed using Image J software (Version 1.38e, NIH, Bethesda, MD).

### Construction of PPI network

The intersection targets were integrated into the STRING database (https://string-db.org/) to obtain information about the protein-protein interaction (PPI) network. We established screening parameters for the organism as “*Homo sapiens*” with a minimum required interaction score of “highest confidence (0.9)”. Subsequently, the resulting PPI information was observed using Cytoscape, before constructing a PPI network.

Using Cytoscape, various parameters of individual nodes in network diagrams, such as degree centrality (DC), betweenness centrality (BC), closeness centrality (CC), and average shortest path length (ASPL) can be calculated to allow a comprehensive analysis of node characteristics in an interaction network. The identification of hub targets for the autophagy-related genes of CRC was accomplished by selecting nodes with DC, BC, and CC values exceeding the corresponding median values in the PPI network.

### Machine learning feature assessment

Selected features were examined using six machine-learning algorithms, i.e., Random Forests (RF), Support Vector Machines (SVM), eXtreme Gradient Boosting (XGB), K Nearest Neighbors (KNN), Naive Bayes (NB) and Linear Discriminant Analysis (LDA). The prediction was performed using the mlr3 package (version 0.16.1) from the R language. To evaluate the performance of selected features in the TCGA-COAD/READ cohort, 10-fold cross-validation generated six models where we obtained the area under the curve (AUC) for the receiver operating characteristic (ROC).

### Statistical analysis

All experiments were randomized and blinded. Data from 3 independent experiments were expressed as Mean ± SD. Statistical analysis was performed using the GraphPad Prism 6.0 software (San Diego, CA, USA). One-way ANOVA followed by Dunnett’s post hoc test was used to compare more than two groups of data. One-way ANOVA, non-parametric Kruskal–Wallis test, followed by Dunn’s post hoc test were used to compare multiple independent groups. P values of < 0.05 were considered statistically significant. Post-tests were run only if F achieved *P* < 0.05 and no significant variance in data homogeneity. The RNA-sequencing database was acquired from the TCGA-COAD/READ database (The Cancer Genome Atlas Research Network). TIL abundance was calculated by applying RNA-seq data to TIP (Tracking Tumor Immunophenotype) [24].

## Results

### ATG7 overexpression in MSI-H/dMMR CRC and correlation with poor prognosis

**Fig. 1:**
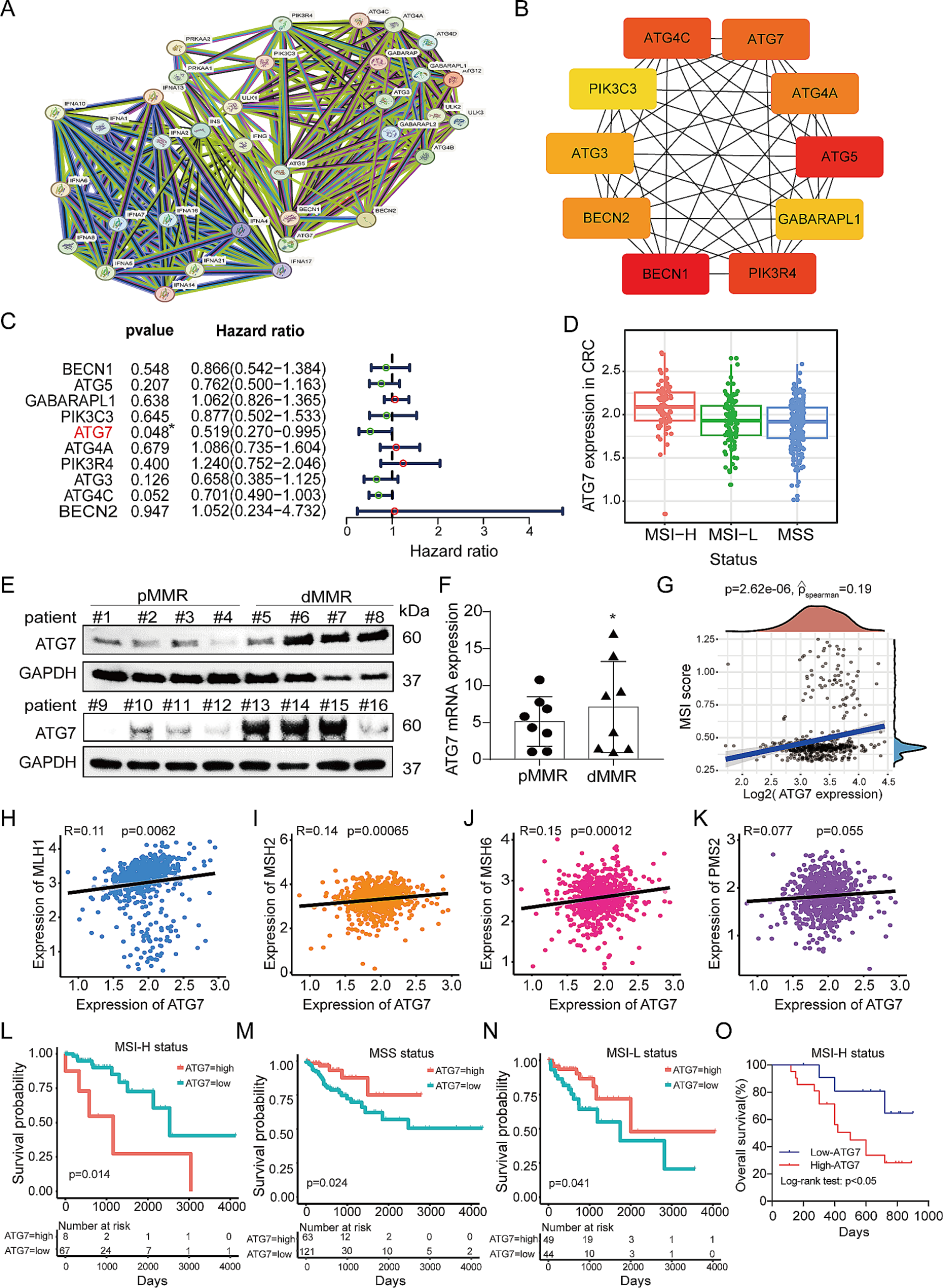
ATG7 is overexpressed in MSI-H/dMMR CRC and correlates with poor prognosis. (A) PPI network of autophagy targets generated by STRING. (B) The top ten hub genes were identified by cytoscape. (C) Univariate analysis of genes associated with OS of patients with CRC in the TCGA. (D) Expression levels of ATG7 in different subtypes of colorectal cancer in TCGA. (E) Protein levels of ATG7 in proficient mismatch repair (pMMR) and deficient mismatch repair (dMMR) tumor tissues were detected by western blot. (F) mRNA levels of ATG7 in pMMR and dMMR tumor tissues were detected by qRT-PCR. (G) The correlation of ATG7 expression and MSI score in TCGA COAD/READ cohort. The correlation analysis between ATG7 and MLH1 (H), MSH2 (I), MSH6 (J), PMS2(K) in TCGA COAD/READ cohort. (L-N) Survival analysis showing the relationship between ATG7 level and the OS of patients diagnosed with MSI-H, MSS or MSI-L CRC in TCGA. (O) Kaplan-Meier analysis was performed to assess the prognostic value of ATG7 in MSI-H CRC in Huashan COAD/READ cohort. Data are presented as the mean ± SD. *P < 0.05, **P < 0.01, ***P < 0.001

PPI analysis was performed for 35 autophagy-related genes on data in the STRING database to identify the promising targets of autophagy in human CRC (Fig. 1A). The resulting TSV data were imported into Cytoscape, which generated a network of 10 hub genes. The color intensity was directly proportional to the degree value and target probability (Fig. 1B). To further explore the prognostic value of these genes in human CRC, the Cancer Genome Atlas (TCGA) database was analyzed to assess the correlation between their expression level and survival time in colorectal cancer patients. The results confirmed that patients with high ATG7 expression had a better overall survival (OS) (Fig. 1C). Further, we analyzed ATG7 levels in different subtypes of colorectal cancer and found ATG7 as the most highly expressed in MSI-H CRC patients (Fig. 1D). Similar results were confirmed by Western blot and qRT-PCR in dMMR CRC patients (Fig. 1E, F). The expression levels also showed that MSI cell lines (HCT-116, LoVo) were higher than microsatellite stable (MSS) cell lines (SW480, SW620) (Fig. S1A, B).

To further analyze the role of ATG7 expression in human dMMR CRC, we conducted a TCGA analysis of the MSI score or the MMR proteins and ATG7. The results revealed that the upregulated ATG7 expression significantly correlated with MSI score (Fig. 1G). The MMR proteins including MutL protein homologue 1 (MLH1), MutS protein homologue 2 (MSH2), and MutS protein homologue 6 (MSH6) positively correlated with ATG7 except postmeiotic segregation increased 2 (PMS2) (Fig. 1H-K). We further examined the relationship between ATG7 expression and survival outcomes in different subtypes of CRC in TCGA. The upregulated of ATG7 expression was associated with poorer survival in CRC patients diagnosed with MSI-H status, unlike the outcomes observed in those diagnosed with MSS or MSI-L status (Fig. 1L-N). We analyzed our samples and found consistent results, implying that patients with high ATG7 expression have a poorer prognosis (Fig. 1O). With this evidence, ATG7 is highly expressed in MSI-H/dMMR CRC and is linked to poor prognosis in patients with MSI-H/dMMR CRC.

### Targeting ATG7 inhibits tumor growth and enhances the infiltration of CD8^+^ T cells

Recent studies developed ATG7-IN-1, a small-molecule compound identified as a selective, and potent ATG7 inhibitor [25]. This compound was used to assess the ATG7 functional role in CRC in vitro. ATG7 inhibition did not exert a significant effect on cell proliferation in LoVo and HCT-116 human CRC cell lines, as well as MC38 mouse CRC cell line (Fig. S1C-F).

**Fig. 2:**
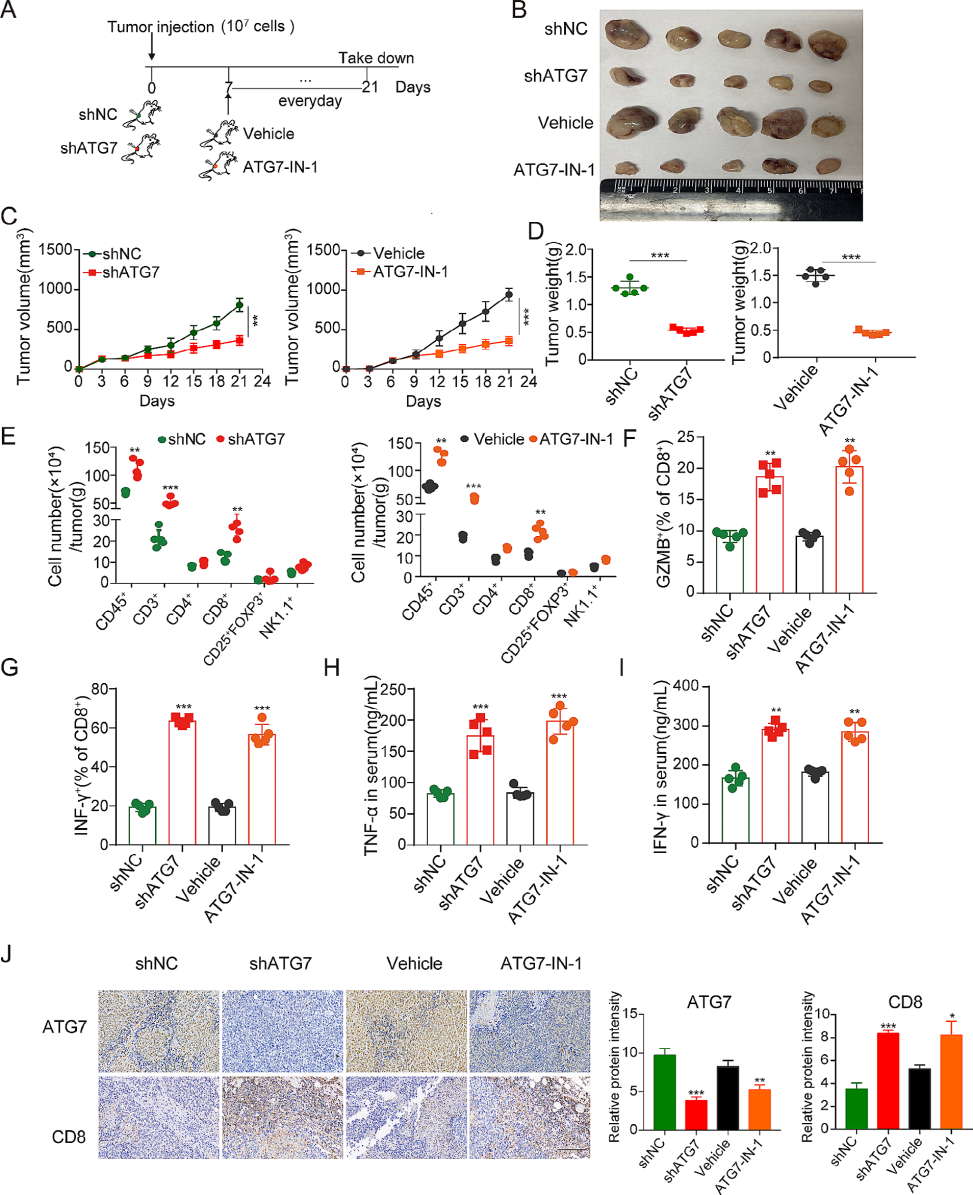
Targeting ATG7 decrease tumor growth and enhance the infiltration of CD8^+ ^T cells. (A) Experimental schedule of shNC, ATG7-targeted (shATG7) or treated with the vehicle or ATG7 inhibitor (ATG7-IN-1) of MC38 colorectal cancer. (B) Image of isolated tumors derived from immunocompetent C57BL/6 mice (*n* = 5). (C-D) Tumor growth curves and tumor weight in immunocompetent C57BL/6 mice. (E) The number of tumor-infiltrating immune cells labeled on the surface of control (shNC) or ATG7-targeted (shATG7) MC38 colorectal tumors or MC38 colorectal tumors treated with control vehicle (vehicle) or ATG7 inhibitor (ATG7-IN-1) by using flow cytometry. Quantification of GZMB^+^CD8^+^ (F) and IFN-γ^+^CD8^+^ (G) cells in MC38 xenografts. (H-I) The TNF-α and IFN-γ level in serum of MC38 xenografts were determined by ELISA analysis. (J) Representative images of immunohistochemical staining for ATG7 and CD8 protein expression patterns in MC38 tumors. (400×magnification). Data are presented as the mean ± SD. *P < 0.05, **P < 0.01, ***P < 0.001

The MC38 cells were subcutaneously inoculated into C57BL/6 mice for in vivo investigation (Fig. 2A). The tumors generated after ATG7 inhibition in immunocompetent C57BL/6 mice exhibited significantly reduced size unlike those treated with control (Fig. 2B). The results in Fig. 2C and D indicate a substantial reduction in tumor growth and weight upon gene targeting of ATG7. To examine whether the ATG7-dependent anti-tumor activity is linked to immune landscape regulation, flow cytometry analysis was carried out to assess immune cell populations within tumors (Fig. S2). We noted a similarly significant increase in the infiltration of viable CD45^+^ cells, CD3^+^ T cells, and CD8^+^ T cells in both shATG7 and ATG7-IN-1 treated MC38 tumors compared to shNC and vehicle control (Fig. 2E). In parallel, we also constructed a mouse model using SW620 cells to further investigate the role of ATG7. Contrary to the results obtained from the MC38 cells, we did not observe any significant changes in CD8^ +^ T cell infiltration in the tumors derived from the SW620 cells (Fig. S3). Additionally, genetic or pharmacological ATG7 inhibition significantly stimulated CD8^+^ T cells to secrete granzyme B (GZMB) and interferon-gamma (IFN-γ) (Fig. 2F and G). The treatment promoted the production of T cell effector cytokines tumor necrosis factor-α (TNF-α) and IFN-γ (Fig. 2H and I). Immunohistochemistry (IHC) staining was also used to confirm the increased infiltration of CD8^+^ cells with ATG7 knockdown (Fig. 2J). The results revealed that whereas ATG7 inhibition did not suppress CRC cell proliferation, it effectively caused an immune response for targeted tumor clearance in vivo, thereby highlighting the key role of CD8^+^ T cells in regulating the observed anti-tumor immunity in ATG7-deficient CRC. Additionally, no apparent signs of toxic side effects including decreased activity, appetite loss, lethargy, and organ damage were observed in mice treated with ATG7-IN-1 (Fig. S4A-4 C).

### ATG7 inhibition mediates anti-tumor effect based on CD8^+^ T cells

**Fig. 3:**
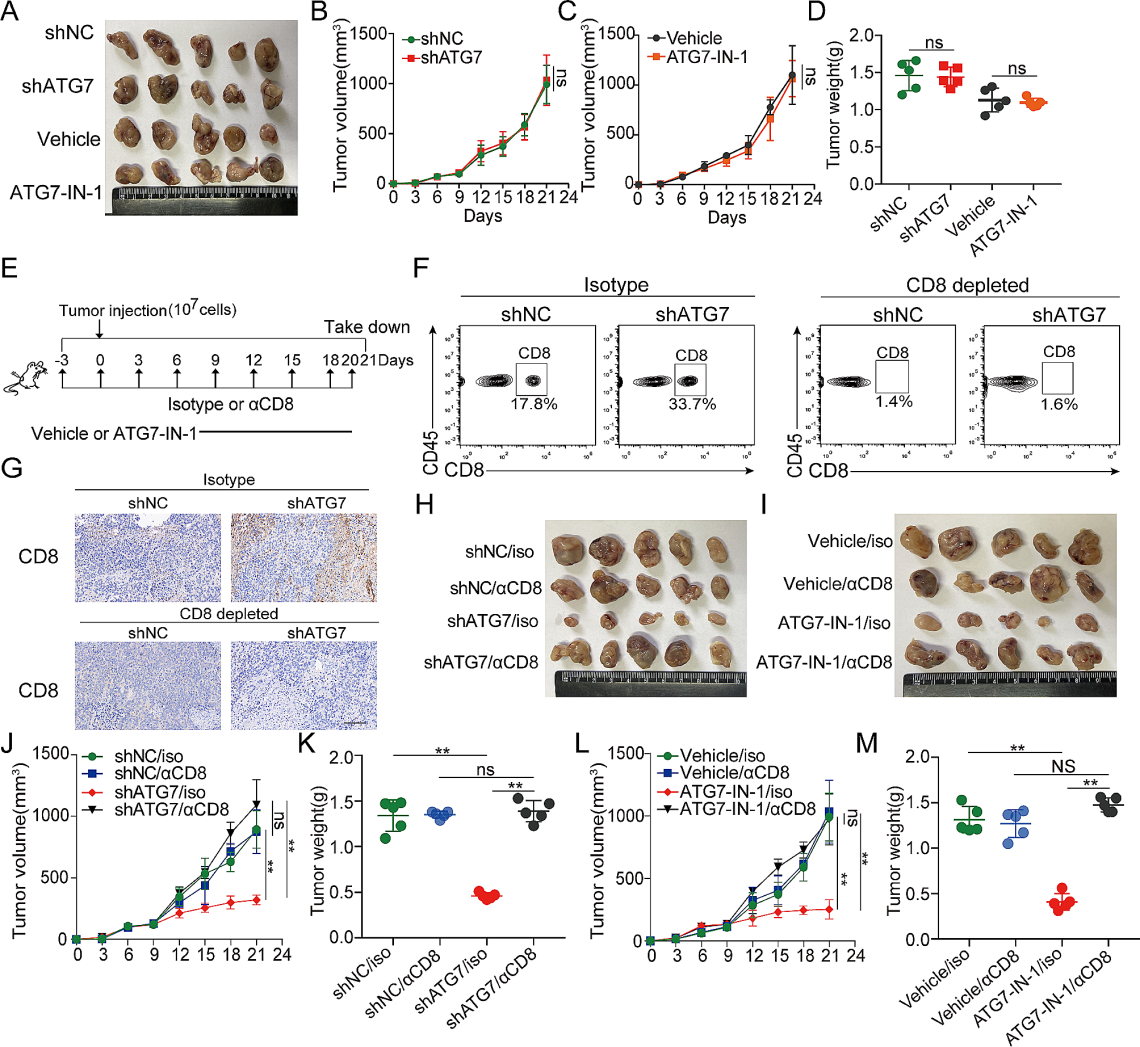
The suppression of colorectal tumor growth targeting ATG7 is contingent upon the presence of CD8^+ ^T cells. (A) Image of isolated tumors derived from immunodeficient nude mice (*n* = 5). 6-week-old male BALB/c nude mice were randomly divided into four groups: shNC group, shATG7 group, vehicle group and ATG7-IN-1 group. Mice were subcutaneously injected with 1 × 10^7^ MC38 cells transfected with lentiviral vectors or control cells. When the tumor volumes reached 100 mm^3^, vehicle group and ATG7-IN-1 group were treated with ATG7-IN-1 (10 mg/kg) or vehicle solution once a day. Tumor tissues were harvested for weight measurement at day 21. (B-C) Tumor volumes were monitored every 3 days. (D) Weight of tumors was measured at time of sacrificed. (E) Schematic representation of the CD8 depletion strategy in shNC, shATG7-treated MC38 tumor-bearing mice or using ATG7-IN-1 to treat. Mice were injected intraperitoneally (i.p.) with 100 µg of either control isotype or αCD8 antibody on the indicated days (black arrows). Then mice were randomly divided into four groups: shNC group, shATG7 group, vehicle group and ATG7-IN-1 group. 1 × 10^7^ MC38 cells were injected into syngeneic C57BL/6 mice and palpable tumors appeared on day 7. Tumor bearing mice were treated with either vehicle or ATG7-IN-1 daily from day 8 to day 20. At day 8, the efficiency of the CD8 depletion was determined by flow cytometry analysis on blood samples of mice chosen randomly. (F) Flow cytometry analysis of CD8^+^ T cells in tumors treated with shNC, shATG7 or anti-CD8. (G) CD8 protein expression was examined by immunohistochemical analysis (400×magnification). (H) Photograph of xenograft shNC, shATG7-treated MC38 tumors from mice treated anti-CD8. (I) Photograph of xenograft tumors from mice treated with ATG7-IN-1 or vehicle or anti-CD8. (J-M) The curve of tumor growth monitored at different time points and isolated tumor weight. Data are presented as the mean ± SD. *P < 0.05, **P < 0.01, ***P < 0.001

To further assess whether the function of ATG7 is fundamentally driven by the immune response, we investigated its role in immunocompromised mice. The effects of shATG7 and ATG7-IN-1 treatment in immunocompromised mice (BALB/c-Nude mice) were examined to assess the effect of adaptive immunity on ATG7-mediated tumor growth and tumor weight reduction. The results revealed that genetic targeting or pharmacological inhibition of ATG7 had no discernible effect on either tumor growth or tumor weight in nude mice (Fig. 3A-D). Subsequently, we investigated whether CD8^+^ T cells regulate ATG7-mediated anti-tumor immune response. CD8^+^ T cells were depleted by αCD8 monoclonal antibody in C57BL/6 mice inoculated subcutaneously with MC38 cells (Fig. 3E). The depletion of CD8^+^ T cells in the tumor was confirmed by flow cytometry and immunohistochemistry (Fig. 3F and G). Treatment with an αCD8 monoclonal antibody (mAb) significantly eliminated the difference in tumor burden between the shNC and shATG7 or vehicle and ATG-IN-1 groups (Fig. 3H-M). These findings indicate that CD8^+^ T cells are primary immune effector cells orchestrating the immune response against colorectal cancer deficient in ATG7.

### ATG7 inhibition restores MHC-I levels in CRC cells

**Fig. 4:**
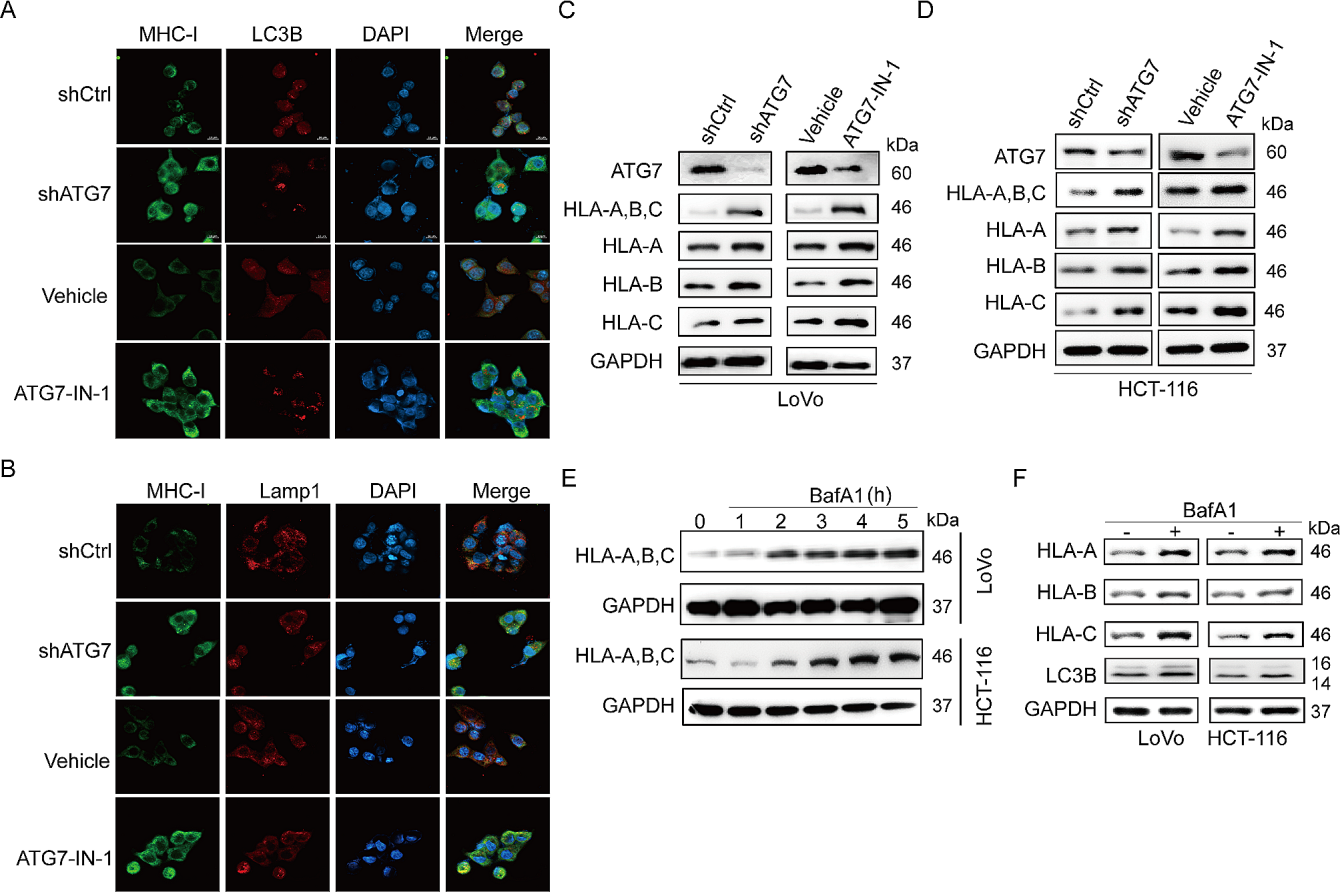
ATG7 inhibition restores MHC-I levels in CRC cells. (A) Localization of MHC-I (green) relative to LC3B (red) positive autophagosomes following ATG7 inhibition in LoVo cells. (B) Localization of MHC-I (green) relative to LAMP1 (red) positive lysosomes following ATG7 inhibition in LoVo cells. Effect of targeting ATG7 on HLA-A, B,C levels in LoVo cells (C) and HCT-116 cells (D) by western blot analysis. (E) Treatment of CRC cells with 150 nM Bafilomycin A1 (BafA1), a lysosomal V-ATPase inhibitor, for the indicated time causes an increase in MHC-I. (F) Effect of BafA1 treatment on expression levels of MHC-I molecules

Lysosomal proteolysis promotes immune evasion of tumor cells by degrading tumor antigens and MHC-I in pancreatic cancer [26]. Therefore, we investigated whether MHC-I is involved in a putative mechanism of ATG7-mediated tumor immune evasion. In LoVo cells, a substantial fraction of the MHC-I colocalized with LC3B, an autophagosome marker, indicating higher autophagy levels in CRC. Furthermore, the immunofluorescence staining revealed that ATG7 suppression induces increased membrane surface MHC-I levels in CRC cells (Fig. 4A). The immunofluorescence staining had cytoplasmic distribution of MHC-I which colocalized with lysosome marker LAMP1. A higher MHC-I expression was observed in ATG7-deficient CRC cells (Fig. 4B). In most species, MHC-I heavy chains are encoded by three polymorphic genes (HLA‑A, HLA‑B, and HLA‑C in humans), constituting the most unique characteristic of MHC molecules [27]. The protein levels determined by western blot showed that ATG7 inhibition upregulated MHC-I, HLA-A, HLA-B, and HLA-C (Fig. 4C, D). We also carried out tests in the MSS cell line and discovered contrary results. Suppressing ATG7 resulted in the inhibition of MHC-I expression (Fig. S5). Moreover, BafA1, a lysosome inhibitor, was used to assess the changes in MHC-I protein levels. The upregulated expression of MHC-I was hinged on the prolonged duration of BafA1 utilization (Fig. 4E). We further confirmed lysosomal inhibition with BafA1 increasing MHC-I subtype proteins (Fig. 4F). These results collectively suggest a specific role for ATG7 in the trafficking of MHC-I to the lysosome.

### ATG7 inhibition promotes MHC-I upregulation in MSI CRC cells via the ROS/ NF-κB signaling pathway

**Fig. 5:**
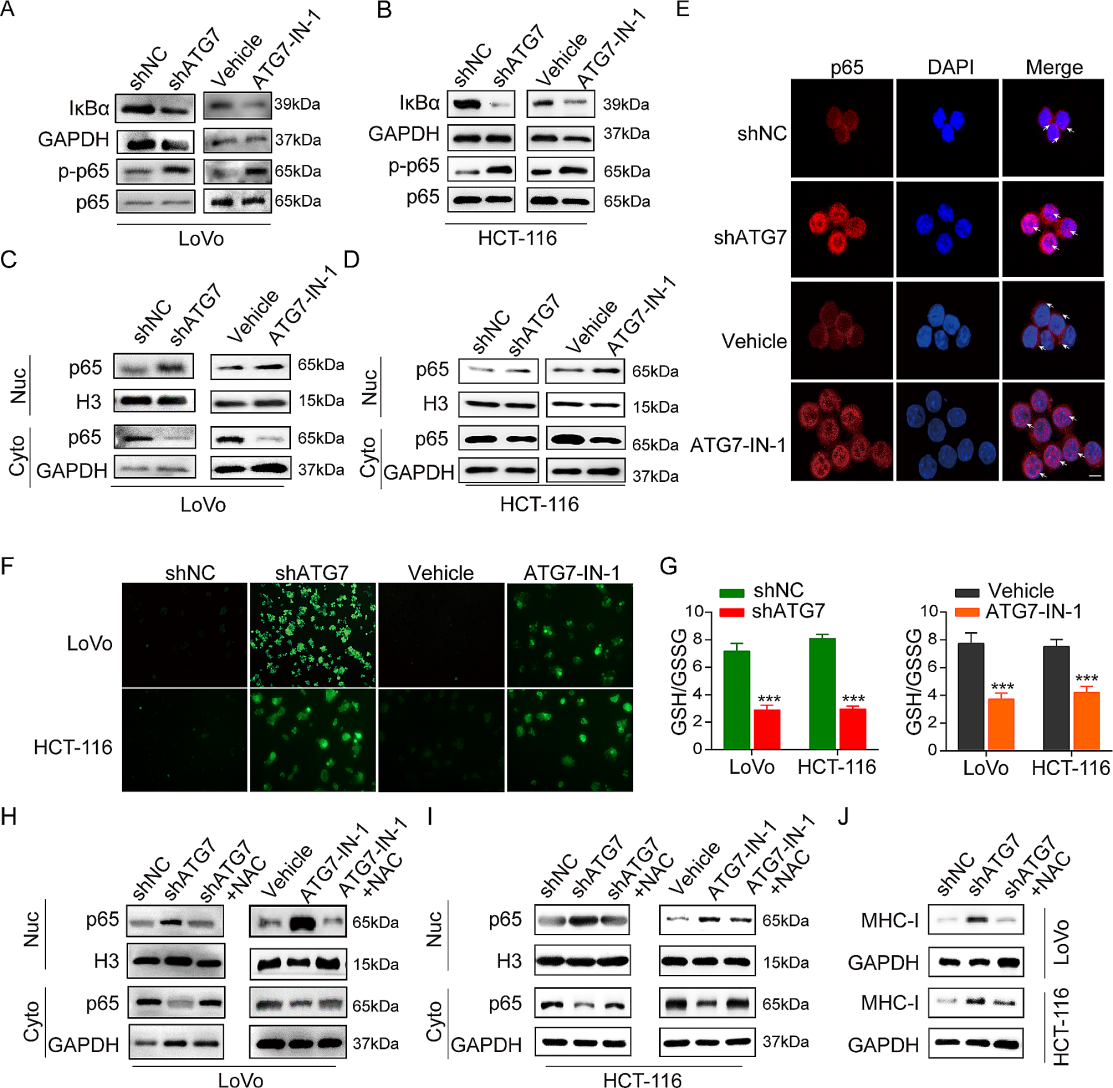
ATG7 inhibition induces MHC-I expression via the ROS/ NF-κB signaling pathway. (A-B) Effect of ATG7 on the protein expression or phosphorylation of NK-κB signaling components in LoVo and HCT-116 cells with shATG7 and ATG7-IN-1 (10 µM) treatment. (C-D) The CRC cells were treated with ATG7 inhibition (shATG7 or ATG7-IN-1, 10 µM, for 24 h), and the expression of p65 protein in both the nucleus and cytoplasm was assessed using western blot analysis. (E) Immunofluorescent staining detected the nuclear translocation of p65 in LoVo cells treated with ATG7 inhibition (shATG7 or ATG7-IN-1, 10 µM) (red: p65 positive stain, blue: nuclei positive stain, the arrows indicate the location of p65). (F) Intracellular ROS (green fluorescence) as detected by DCFH-DA staining (green). CRC cells were treated with ATG7 inhibition for indicated times and fluorescence images were captured. (G) GSH/GSSH ratio was measured in MSI CRC cells with shATG7 and ATG7-IN-1 treatment. (H-I) The impact of ATG7 inhibition (shATG7 or ATG7-IN-1, 10 µM) or NAC treatment (10 mM) for 1 h on the levels of p65 protein in both the nucleus and cytoplasm of CRC cells was evaluated. (J) Effect of ATG7 inhibition (shATG7 or ATG7-IN-1, 10 µM) or NAC treatment (10 mM) on MHC-I expression in CRC cells was determined by western blot. Data are presented as the mean ± SD. *P < 0.05, **P < 0.01, ***P < 0.001

We then investigated the underlying mechanisms involved in the in vitro up-regulation of MHC-I after ATG7 targeting using shATG7 or ATG7-IN-1. One previous study revealed that NF-κB activation potentiates cancer chemoimmunotherapy by inducing MHC-I [28]. As depicted in Fig. 5 (A and B), we noted a decrease in IκBα and an increase in p-p65 following ATG7 inhibition, confirming NF-κB signaling pathway activation. We further analyzed the nuclear translocation of p65 in CRC cells treated with shATG7 and ATG7-in-1, revealing a marked occurrence of p65 translocation from the cytoplasm to the nucleus via ATG7 inhibition (Fig. 5C-E).

As a secondary messenger, reactive oxygen species (ROS) modulates various carcinogenic signaling pathways during cancer progression and is generated as a byproduct of mitochondrial oxidative phosphorylation [29]. We investigated whether ATG7 modulates ROS levels. After suppressing ATG7 in LoVo cells and HCT-116, ROS levels were evaluated by DCFH-DA staining. Results showed that ATG7 inhibition significantly increases ROS levels (Fig. 5F). Additionally, treatments with shATG7 and ATG7-IN-1 significantly decreased the GSH/GSSG ratio within CRC cells (Fig. 5G), indicating that ATG7 inhibition changes the redox homeostasis in CRC cells by causing oxidative stress.

Recent studies have reported that ROS is an important upstream factor of the NF- κB signaling pathway [30, 31]. Therefore, our hypothesis posits that increased levels of reactive oxygen species (ROS) may activate the NF-κB signaling pathway by targeting ATG7 in CRC cells. To validate this conjecture, we assessed the relationship between augmented ROS levels and NF-κB signaling activation in CRC cell lines. Western blot analysis revealed that treatment with ROS scavengers (NAC) effectively blocked p65 translocations induced by the ATG7 inhibitor (Fig. 5 (H and I)). We further evaluated the role of ATG7 inhibition-mediated ROS/NF-κB signaling in regulating MHC-I expression. ATG7 inhibitor-mediated induction of MHC-I hyperexpression was reversed by NAC (Fig. 5J).

We evaluated the expression changes in the NF-κB signaling pathway within MSS cells. It suggested that the suppression of ATG7 does not lead to significant alterations in the levels of IκB and p-p65 in SW480 and SW620 cells (Fig. S6A and B). We further examined the levels of ROS in MSS cells. Both DCFH-DA fluorescence and GSH/GSSG ratio revealed no significant changes after inhibiting ATG7 in the ROS levels (Fig. S6C, D).

Collectively, these findings suggest that the ROS/NF-κB signaling pathway plays a crucial role in regulating MHC-I expression in MSI CRC cells via targeted ATG7 modulation.

### Targeting ATG7 improves the therapeutic benefit of anti-PD-1

**Fig. 6:**
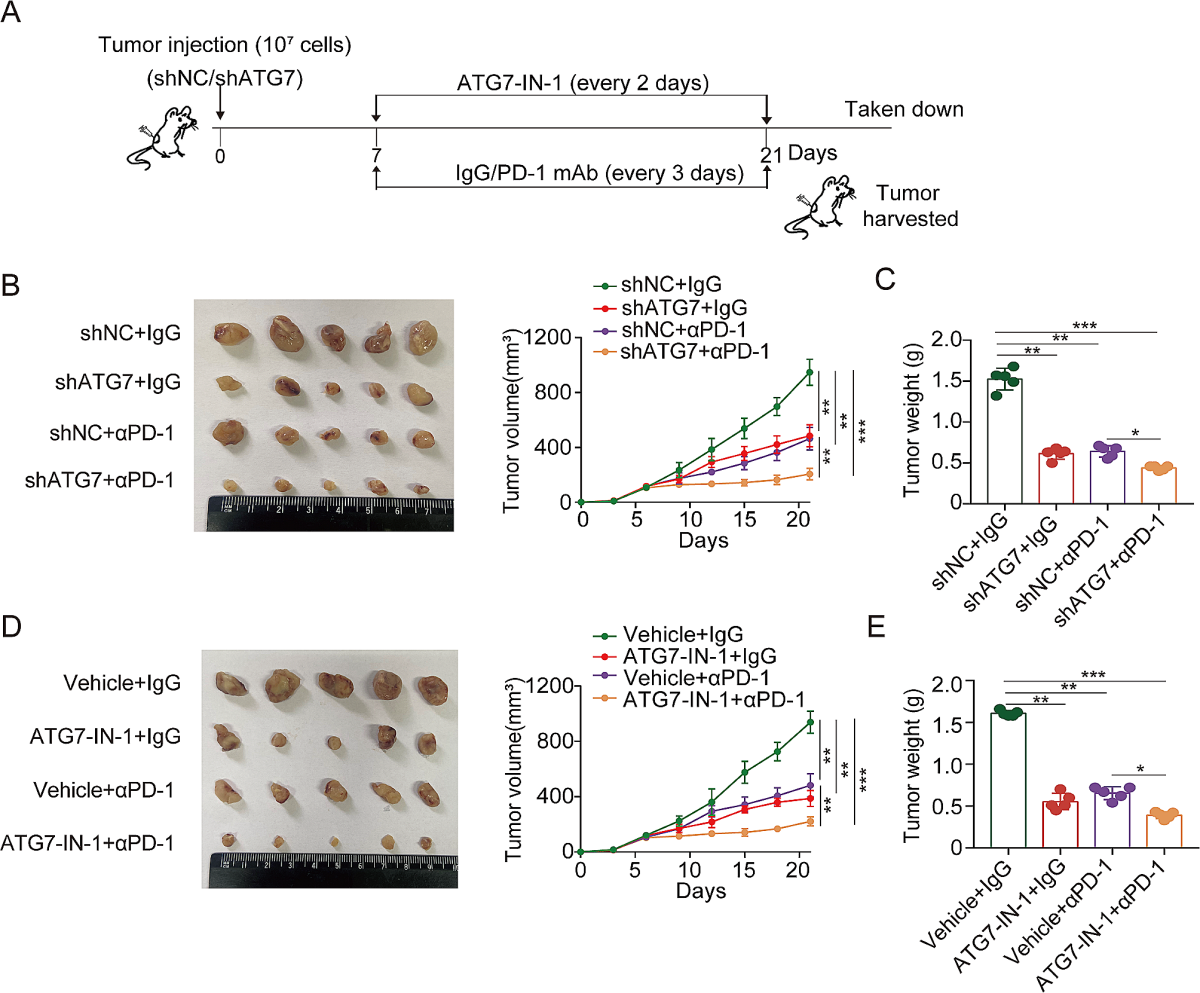
Targeting ATG7 enhances the efficacy of anti–PD-1 therapy. (A) Schematic diagram of the drug intervention protocol utilizing the ATG7 inhibition and/or anti-PD-1 antibody to treat C57BL/6 mice. Image of isolated tumors and tumor growth curves (B, D) and tumor weight (C, E) of MC38 xenografts treated with control (shNC, vehicle) or ATG7 inhibition (shATG7, ATG7-IN-1) combined with anti-PD-1

Considering that CD8^+^ T cell-dependent metabolic remodeling, facilitated by ATG7 inhibition, improves the efficacy of anti-tumor immunity, we investigated whether targeting ATG7 could further improve anti-tumor efficacy of PD-1 blockade (Fig. 6A). Our findings confirmed that treatment with shATG7 or ATG7-IN-1 alone suppressed tumor growth and weight in the MC38 mouse model, which has been identified as an immunological hot tumor responding to anti-PD-1 [32]. As expected, we detected the effect of anti-PD-1 monoclonal antibody treatment on tumor volume and tumor weight. Importantly, the combination therapy targeting ATG7 and anti-PD-1 demonstrated improved treatment efficacy unlike the monotherapy approach (Fig. 6B-E). Summarily, the results provide strong evidence that ATG7 improves the responsiveness of Mc38-bearing mice to anti-PD-1.

### HMGCR reduction causes less cholesterol accumulation with a synergistic effect on ATG7-deficient inducing anti-tumor efficacy of anti-PD-1

**Fig. 7:**
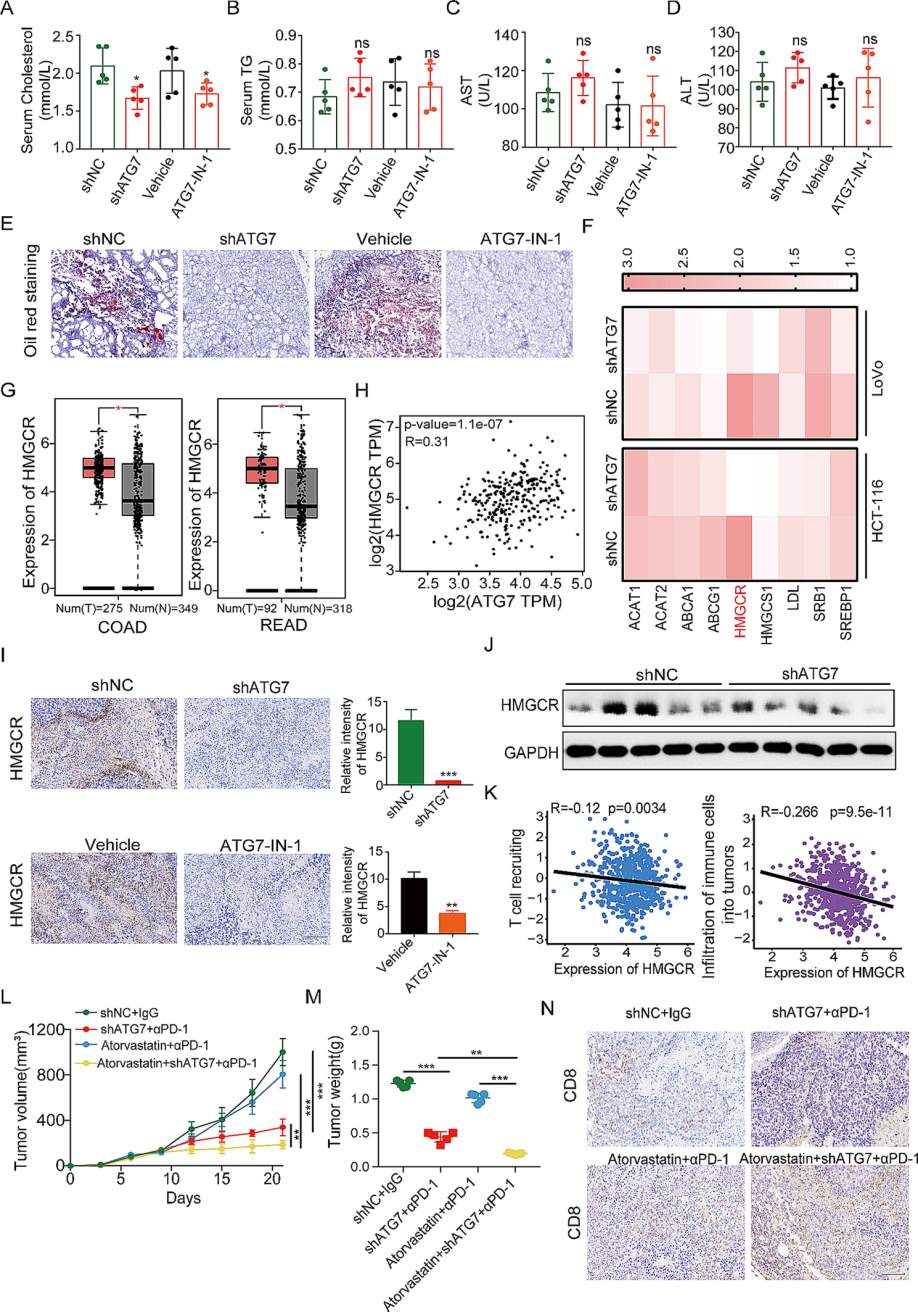
HMGCR-induced cholesterol accumulation contributes to the role of ATG7 in colorectal cancer. (A-D) Serum cholesterol, TG, ALT and AST levels of MC38 tumor mice. (E) Representative oil red staining for tumor tissues (400×magnification). (F) The mRNA expression levels of the key genes of cholesterol metabolism upon treatment of ATG7 inhibition in CRC cells were measured by qRT-PCR. (G) The expression of HMGCR in TCGA COAD/READ cohort identified by GEPIA2. (H) The correlation between ATG7 and HMGCR in TCGA COAD/READ cohort. (I) IHC staining with antibody against HMGCR and quantification of relative intensity of HMGCR staining in MC38 xenografts treated with ATG7 inhibition (400×magnification). (J) The HMGCR protein level in shATG7-MC38 xenografts was analyzed by western blot. (K) TIP analysis was used to determine the correlation between HMGCR and T cell recruiting or infiltration of immune cells. (L-M) Tumor growth curves (L) and tumor burdens (M) of MC38 xenografts treated with shNC, shATG7 or atorvastatin combined with IgG or anti–PD-1 mAb (*n* = 5). (N) CD8 protein expression was examined by immunohistochemical analysis (400×magnification). Data are presented as the mean ± SD. *P < 0.05, **P < 0.01, ***P < 0.001

Cholesterol accumulation caused by SQLE depletion exacerbates the progression of colorectal cancer by activating the β-catenin oncogenic pathway and deactivating the p53 tumor suppressor pathway [33]. Therefore, our hypothesis posited that ATG7 inhibition to impede cholesterol accumulation would cause anti-tumor immune response activation. Upon collating clinical data from patients, we noted a positive correlation between upregulated ATG7 expression and increased total cholesterol (TC) levels (Table S2). We further measured the serum levels of TC, TG, ALT, and AST in CRC mice and discovered that ATG7 inhibition could significantly decrease TC accumulation (Fig. 7A-D). Oil red staining also showed that ATG7 blocking could effectively suppress lipid accumulation (Fig. 7E).

To further elucidate the proteins involved in cholesterol metabolism, we evaluated the mRNA expression levels of the genes after ATG7 inhibition in CRC cells. Unlike other genes, HMGCR exhibited the most downregulated expression (Fig. 7F). TCGA revealed that HMGCR expression is greater in human CRC than in normal specimens (Fig. 7G). We subsequently searched the TCGA database and discovered a positive correlation between HMGCR and ATG7 in CRC using GEPIA 2.0 (Fig. 7H). We then validated the correlation between HMGCR and ATG7 in vivo by performing IHC staining, and Western blot on CRC mice. In the ATG7 knockdown groups, we observed a corresponding decrease in the expression of HMGCR (Fig. 7I, J).

Research has shown that inhibiting cholesterol potentially improves immune checkpoint therapy for cancer [34]. Herein, the TIP tool was used to evaluate the relationship between HMGCR expression and immune response in tumors. TIP analysis revealed that the activity of “T cell recruiting” and “Infiltration of immune cells into tumor” were negatively correlated with HMGCR expression (Fig. 7K). Further, we investigated whether HMGCR targeting could improve the ATG7-deficient inducing anti-tumor efficacy of anti-PD-1. In line with previous results, we observed positive effects of anti-PD-1 plus shATG7 treatment on tumor volume and tumor weight. Moreover, the combination of shATG7 and HMGCR inhibitor (atorvastatin) with anti-PD-1 therapy provided additional benefits over the monotherapy strategy (Fig. 7L, M). Tumor samples were further harvested for further investigation. IHC analysis revealed that the increased infiltration of CD8^+^ T cells could promote the better responsiveness observed in the combination treatment group (Fig. 7N). In summary, our data provide solid evidence supporting ATG7 targeting in concert with atorvastatin as a viable treatment strategy to increase the efficacy of anti-PD-1 therapy in CRC.

### The 3-year survival probability of colorectal cancer patients was predicted based on ATG7-HMGCR expression pattern through the utilization of machine learning algorithms

**Fig. 8:**
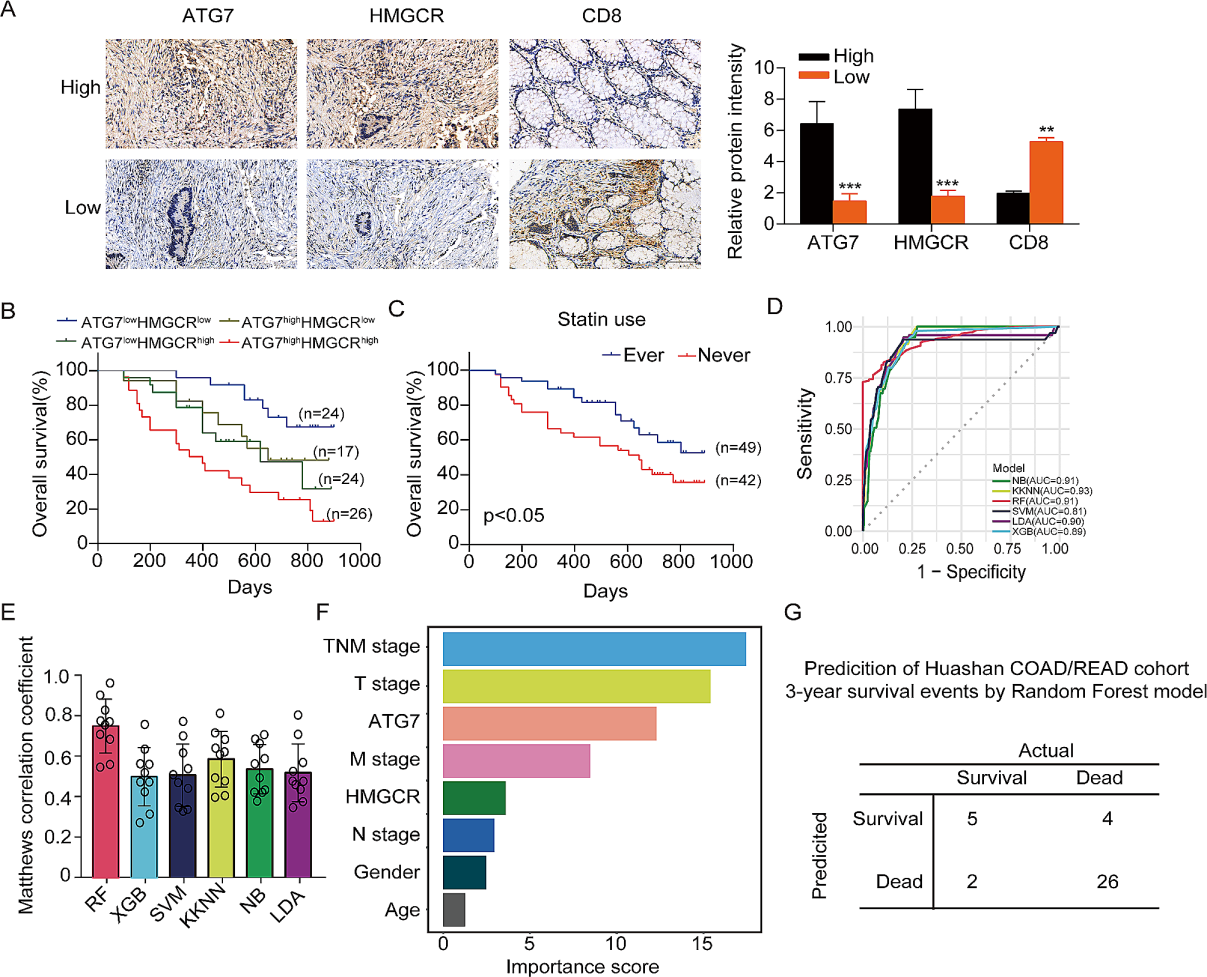
Predicting the 3-year survival probability of colorectal cancer patients using ATG7-HMGCR expression patterns combined with machine learning algorithms. (A) Representative images and quantification of IHC staining for ATG7, HMGCR, and CD8 expression were obtained from 12 clinical samples, which were randomly selected from 6 different groups of CRC patients with varying levels of ATG7 expression. (high: high ATG7 expression group, low: high ATG7 expression group). Scale bars, 50 μm. (B) The prognostic value of combining ATG7 and HMGCR levels was analyzed by Kaplan-Meier analysis in the Huashan COAD/READ cohort. (C) The OS survival analysis of patients who ever or never used statins was analyzed by Kaplan-Meier analysis in the Huashan COAD/READ cohort. (D) ROC curve analysis of six machine learning algorithms for prediction the 3-year survival probability of COAD patients in the TCGA-COAD/READ cohort. (E) Matthews correlation coefficient of six machine learning algorithms. (F) The established random forest model was used to rank the importance of clinical parameters. (G) Prediction of the 3-year survival events for Huashan COAD/READ cohort patients using the established random forest model

To further investigate the expression levels of ATG7, HMGCR and CD8 in CRC patient tissues, we performed immunohistochemical staining on samples from our Huashan cohort. The samples were divided into high and low groups based on the level of ATG7 expression determined by immunohistochemical staining. The findings demonstrated a positive association between ATG7 expression and HMGCR expression, as well as a negative association between ATG7 expression and CD8^+^ T cells infiltration in human CRC tissues (Fig. 8A). Furthermore, high expression levels of ATG7 combined with elevated HMGCR were associated with poor OS of patients (Fig. 8B). Next, we examined the prognosis of CRC patients who had used or never used statin, and the result suggested that patients who had used statins had better OS (Fig. 8C).

In order to further explore the prognostic implications associated with ATG7 and HMGCR expression patterns in colorectal cancer (CRC) patients, we utilized six different machine learning algorithm models to predict the incidence of 3-year survival events. We screened patients from the TCGA-COAD/READ cohort, and selected those who survived beyond 3 years and those who died within this time (totaling 228 patients). The cohort comprised patients who achieved successful 3-year survival (133 patients) and another group with patients who failed to achieve the 3-year survival (95 patients). Next, we collected relevant clinical data and the expression levels of ATG7 and HMGCR for these subjects. Using the mlr3 package in the R programming language, we developed six machine learning algorithm models for predicting the probability of 5-year survival events in CRC patients. A detailed comparison was conducted on the prediction performance of the six models in the TCGA-COAD cohort. The random forest model had the highest performance in predicting 3-year survival events, with an AUC of 0.91 and matthews correlation coefficient of 0.75 (Fig. 8E, F). Therefore, the random forest model was selected as the final prediction model. Based on the feature importance values obtained from the random forest model, the significance of each clinical parameter was determined by aggregating their respective feature importance values. Subsequently, the random forest model was applied to rank the importance of these clinical parameters. It was found that the expression of ATG7 and HMGCR genes was ranked third and sixth positions, respectively, in terms of importance scores in the random forest model (Fig. 8F). To explore the model’s efficacy, patients (*n* = 37) who either survived for more than 3 years or died within 3 years were selected from the Huashan cohort. Subsequently, we utilized a well-established random forest model to predict the 3-year survival outcomes for the selected patients. Comparison of the prediction results with clinical follow-up data revealed a prediction accuracy of 89% in the validation cohort (Fig. 8G).

## Discussion

In this study, we addressed an unmet clinical need by exploring the efficacy of immune checkpoint inhibitors. This study shows that inhibiting ATG7 activates NF-KB/ROS, thereby upregulating MHC-I expression and improving CD8^+^ T cell infiltration and immune response. In addition, we identified HMGCR as a key gene involved in cholesterol metabolism and contributing to the anti-tumor effects of ATG7 inhibition. This implies that combining atorvastatin with ATG7 inhibition may potentiate the benefits of immunotherapy in colorectal cancer patients. Several studies have demonstrated that autophagy inhibition can prevent tumor growth [12, 13, 35]. However, the impact of targeting autophagy on the immune landscape of colorectal cancer is not well understood. We observed a significant increase in ATG7 expression, which correlated with a poorer prognosis in patients with MSI-H/dMMR CRC. This indicates that elevated ATG7 levels might contribute to the reduction in efficacy of immunotherapy in colorectal cancer. Prior research has indicated the involvement of autophagy in the anti-tumor immune response [26, 36]. Evidence has shown that mice with pancreatic tumor carrying a dominant negative ATG4B mutant displayed a significantly higher number of CD68^+^ macrophages in the tumors compared to mice carrying wild-type ATG4B [37]. Moreover, another study showed that CD4^+^ T cells lacking Atg3 or Atg5 can increase IL-9 expression and autophagy inhibition enhanced T helper 9 cell anticancer effects in vivo, and mice with T cell-specific deletion of Atg5 showed decreased tumor outgrowth in an IL-9-dependent manner [38]. Here, we found that genetic targeting of ATG7 or pharmacological inhibition affected the immune landscape, especially that of CD8^+^ T cells. This presents a combination therapy strategy with improved efficacy of immunotherapy in CRC patients.

Recent evidence has shown that MHC-I mediates the recruitment of T cells. Stimulating antigen-exposed psDCs triggered two significant alterations: elevated MHC-I molecules on their surface and increased lipid peroxidation, a signaling process within cells. Administration of the modified psDCs into mice enhanced pathogen-specific CD8 T cell responses, resulting in protection against infection. This protective mechanism was dependent on the activity of CD8 T cells [39]. Depletion of autophagy proteins or treatment with chloroquine (CQ) increases MHC-I expression on the surface of dendritic cells, thereby augmenting CD8^+^ T cell responses in viral infection models [40]. The immunofluorescence staining results revealed enrichment of MHC-I in autophagosomes and lysosomes, suggesting that MHC-I may undergo degradation via the autophagy-lysosome pathway. Moreover, inhibition of ATG7 resulted in increased membrane expression of MHC-I on colorectal cancer cells. Targeting ATG7 or lysosomal inhibition further decreased MHC-I protein levels. Therefore, ATG7 participation in MHC-I trafficking to lysosomes and its inhibition may increase the infiltration level of CD8^+^ T cells in the TME by increasing MHC-I upregulation.

Metabolic processes associated with mitochondria are among the main factors contributing to the generation of endogenous ROS, which regulates cellular signaling [41]. The NF-κB signaling pathway plays a crucial role in mediating the interplay between tumor inflammation, immunity, and response to therapies. This pathway acts as a sensor for ROS, molecules linked to inflammation and stress, and translates these signals into various cellular responses. Notably, research has found a link between low levels of a protein called MHC-I and the ability of tumors to evade immune defenses, leading to poorer outcomes with immunotherapy. Activation of the TLR3/NF-κB pathway significantly upregulated MHC-I gene expression, thereby inhibiting immune escape and augmenting immunotherapy in muscle-invasive bladder cancer. Our results indicated that the NF-κB pathway was activated following ATG7 deletion. Inhibition of ATG7 enhanced MHC-I expression in CRC cells via ROS-NF-KB-dependent mechanism, as confirmed by experiments using ROS inhibitor (NAC).

A recent study showed that inhibition of PCSK9, a key protein in the regulating cholesterol metabolism, significantly increased MHC-I expression and efficacy of immune checkpoint therapy [34]. Inhibition of the cholesterol esterifying enzyme ACAT1 has been shown to promote the anti-tumor effect of CD8 + T cells by facilitating the clustering of T cell receptors [44]. In the present investigation, we observed a positive association between elevated ATG7 expression and cholesterol levels in patients. Notably, HMGCR, a target of statins and a key enzyme that catalyzes the de novo cholesterol synthesis, was found to modulated immune infiltration mediated by ATG7. Furthermore, the combination of statin and ATG7 targeting therapy augmented the anti-PD-1 immunotherapy sensitizing effect of ATG7 inhibition. Additionally, the prognostic value of the ATG7-HMGCR axis in colorectal cancer patients was investigated by evaluating its expression patterns through machine learning algorithms for predicting the 3-year survival probability.

**Fig. 9:**
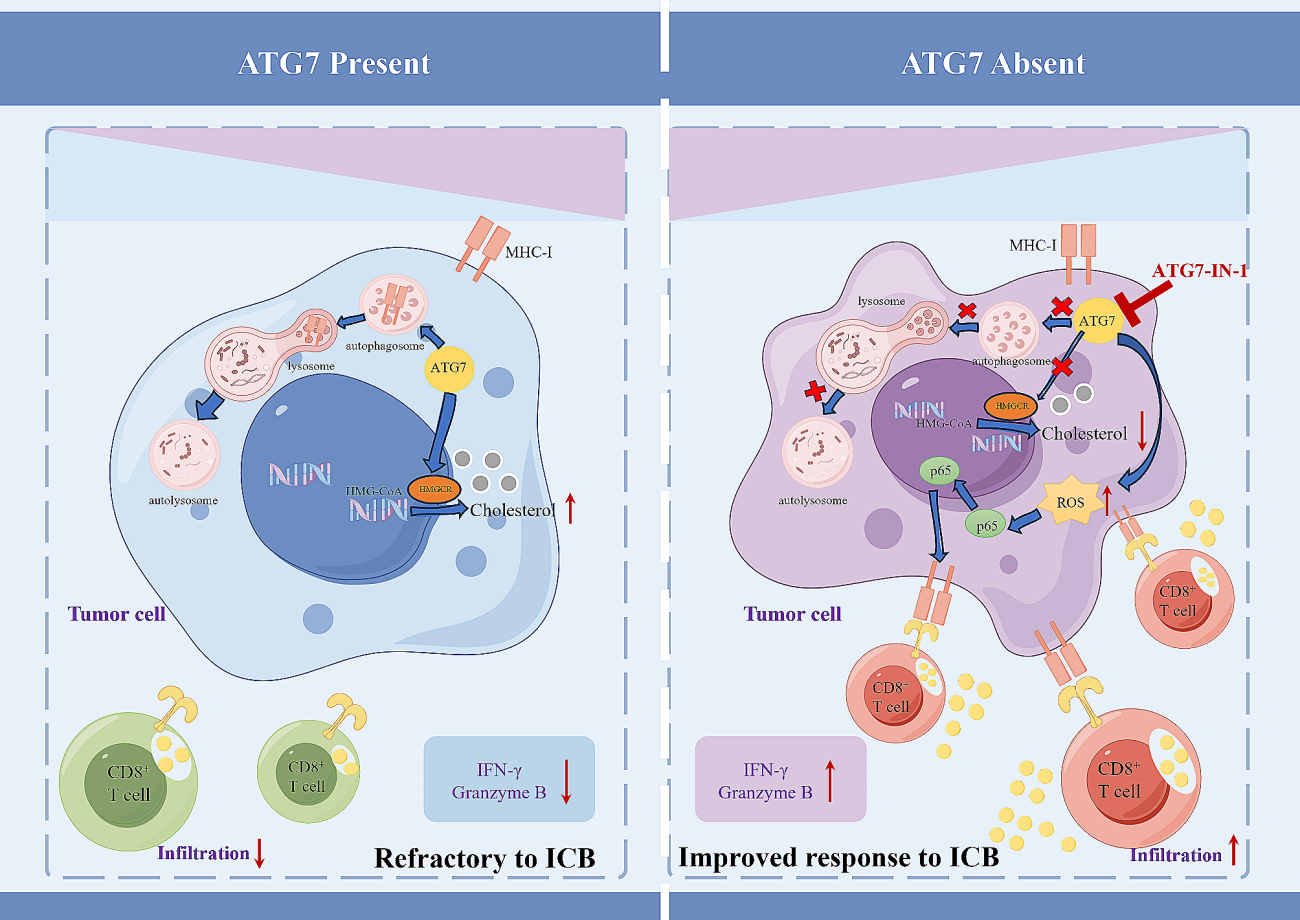
Illustration of the proposed working model

In conclusion, our study explores the intrinsic role of ATG7 in creating an immunosuppressive milieu in CRC (Fig. 9). Inhibition of ATG7 enhanced the anti-tumor immune response in CRC, as evidenced by increased MHC-I expression via the ROS/NF-κB signaling pathway. Furthermore, co-administration of the HMGCR inhibitor statin (atorvastatin) further potentiated the efficacy of anti-tumor immunotherapy. Currently, there are no effective strategies for Improving response rates to immune checkpoint blockade therapies. Our study proposes a promising new approach that combines an ATG7 inhibitor with cholesterol-lowering drugs and immune checkpoint blockade. This innovative combination has the potential to overcome resistance and offer significant benefits for patients with colorectal cancer.

## Conclusions

Taken together, this study suggests that inhibiting ATG7 can activate CD8^+^ T cells, which presents a novel mechanism for colorectal cancer (CRC) immunotherapy. This is mediated by activation of the ROS/NF-κB pathway to upregulate MHC-I expression on the cell membrane. Moreover, we have confirmed that combining ATG7 inhibitor with the HMGCR inhibitor atorvastatin may be an effective targeted combination therapy. Therefore, simultaneous targeting of ATG7 and cholesterol inhibition represents a potential therapeutic strategy for enhancing the responsiveness of MSI-H CRC patients to immunotherapy.

### Electronic supplementary material

Below is the link to the electronic supplementary material.

Supplementary Material 1

## Data Availability

All the presenting data are available within the article or supplementary files.

## Abbreviations

ATG7: Autophagy-related protein 7
CRC: Colorectal cancer
ICB: Immune Checkpoint Blockade
MSI: Microsatellite instability
MHC-I: Major Histocompatibility Complex class I
ROS: Reactive oxygen species
NF-κB: Nuclear factor kappa-B
HMGCR: 3-Hydroxy-3-methylglutaryl coenzyme A reductase
PD-1: Programmed Death-1
MSI-H: High microsatellite instability
dMMR: Deficient mismatch repair
pMMR: Proficient mismatch repair
MSS: Microsatellite stable
PMS2: Postmeiotic segregation increased 2
MLH1: MutL protein homologue 1
MSH2: MutS protein homologue 2
TC: Total cholesterol
TG: Serum cholesterol, triglyceride
ALT: Alanine aminotransferase
AST: Aspartate aminotransferase
