# Supplementary material for: Inhibition of autophagy-related protein 7 enhances anti-tumor immune response and improves efficacy of immune checkpoint blockade in microsatellite instability colorectal cancer
Source: J Exp Clin Cancer Res. 2024 Apr 16;43:114. doi: 10.1186/s13046-024-03023-w (PMC11020677; doi:10.1186/s13046-024-03023-w)

**Additional file 1: Supplementary Fig. 1. The inhibition of ATG7 in vitro does not result in any significant alteration in tumor growth.**

(A-B) ATG7 protein and mRNA levels in CRC cell lines. (C) Cell viability was determined by CCK8 kit after cells were cultured with indicated concentrations of ATG7-IN-1. (D) Colony formation assays of CRC cells treated with ATG7-IN-1 (5 μM, 10 μM).

**Additional file 2: Supplementary Fig. 2. Gating strategy for flow cytometry analysis of lymphoid population in CRC tumors.**

A Live/Dead dye was used to select only live cells. CD45^+^ CD3^-^ NK1.1^+^cells were defined as NK cells. Lymphocytes were defined as the CD3^+^ subpopulation of the CD45^+^ NK1.1^-^gate. CD4^+^ and CD8^+^ T lymphocytes were derived from the CD3^+^ subpopulation. Tregs were subdivided from CD4^+^ T lymphocytes and were defined as Foxp3^+^. CD8^+^ T lymphocytes were further stained for IFN-γ^+^CD8^+^ T cells and GZMB^+^ CD8^+^ T cells.

**Additional file 3: Supplementary Fig. 3.** **Analysis of CD8^+^ T cell infiltration in a mouse model constructed with SW620 cells.**

The model was developed by subcutaneously injecting SW620 cells into mice. The tumors were then harvested and processed for flow cytometry to measure the level of CD8+ T cell infiltration.

**Additional file 4: Supplementary Fig. 4.** **ATG7-IN-1 does not have obvious side effects.**

Body weight curve (A) and weight of final dissected organs (B) of mice after ATG7-IN-1 (10 mg/kg) treatment in MC38 xenografts. (C) H&E stained paraffin sections of organs. Scale bar, 100 μm.

**Additional file 5: Supplementary Fig. 5.** **Impact of ATG7 suppression on MHC-I expression in MSS CRC cell lines.**

Effect of targeting ATG7 on HLA-A, B, C levels in SW480 and SW620 cells by western blot analysis.

**Additional file 6: Supplementary Fig. 6.** **Evaluation of ATG7 suppression effects on NF-κB signaling pathway and ROS levels in in MSS CRC cell lines.**

(A-B) Protein expression or phosphorylation of NK-κB signaling components in SW480 and SW620 cells with shATG7 and ATG7-IN-1 (10 μM) treatment. (C) Intracellular ROS (green fluorescence) as detected by DCFH-DA staining (green). SW480 or SW620 cells were treated with ATG7 inhibition for indicated times and fluorescence images were captured. (D) GSH/GSSH ratio was measured in MSS CRC cells with shATG7 and ATG7-IN-1 treatment.

**Additional file 7: Supplementary Table S1. The** **primer sequences of genes in qRT-PCR assay.**

**Additional file 8: Supplementary Table S2. Clinical characteristics and blood lipid level of patients with different ATG7 expression levels.**

**Supplementary Fig. 1.**


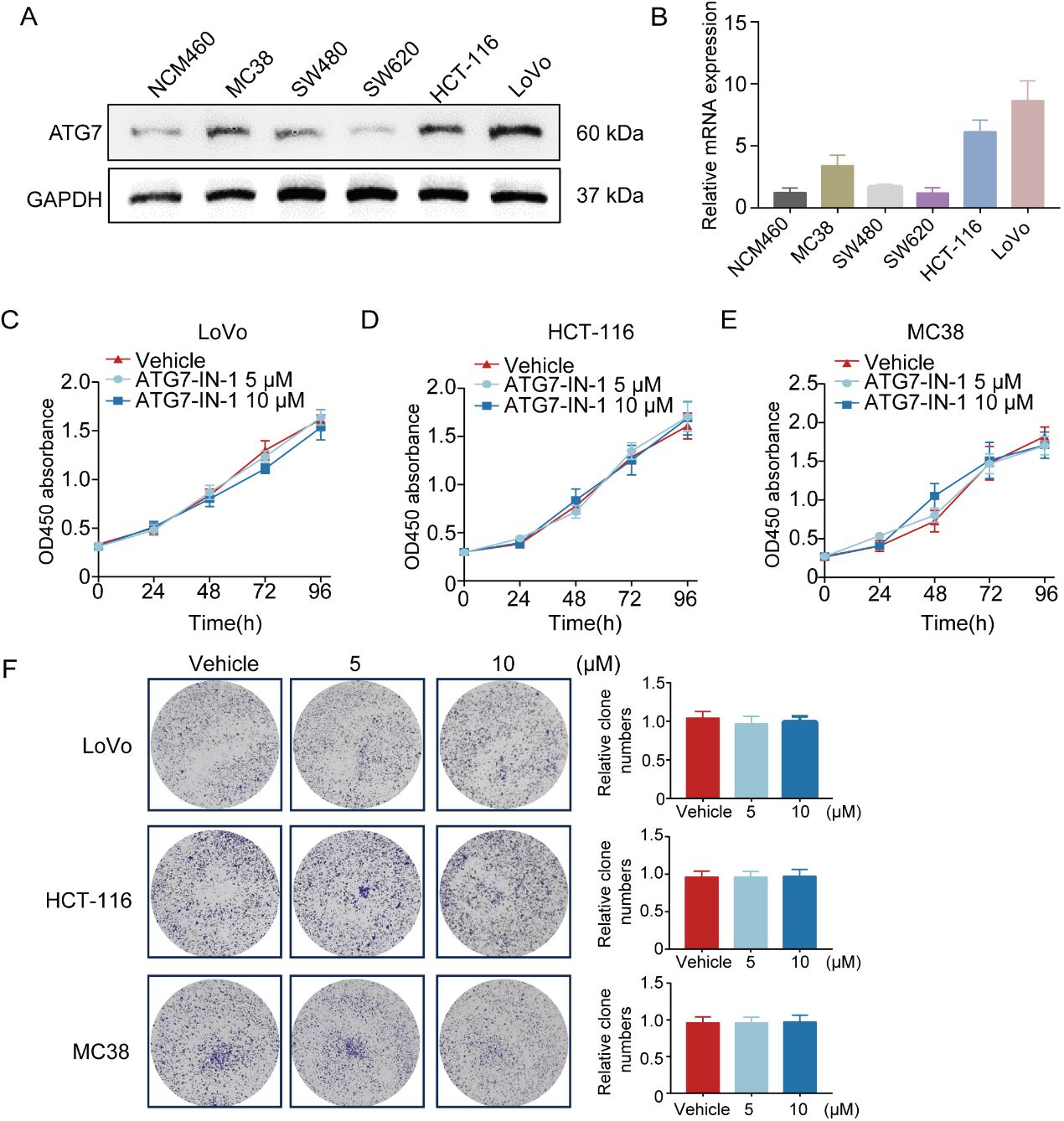


**Supplementary Fig. 2.**


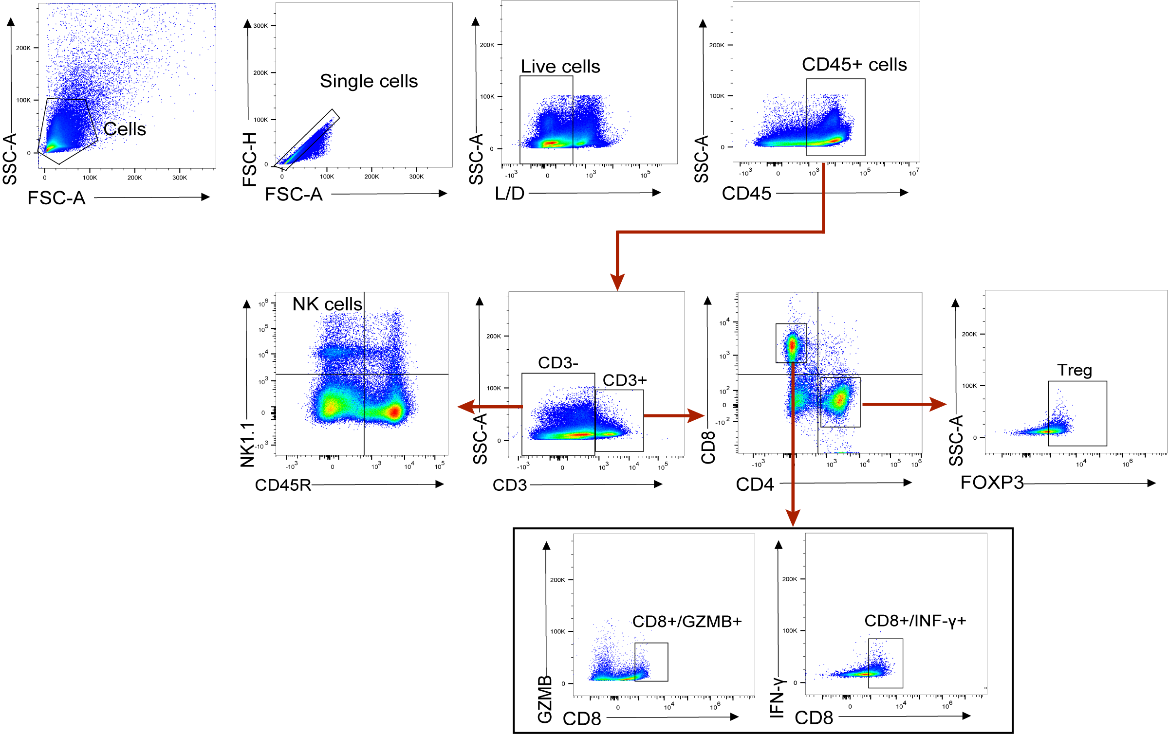


**Supplementary Fig. 3.**


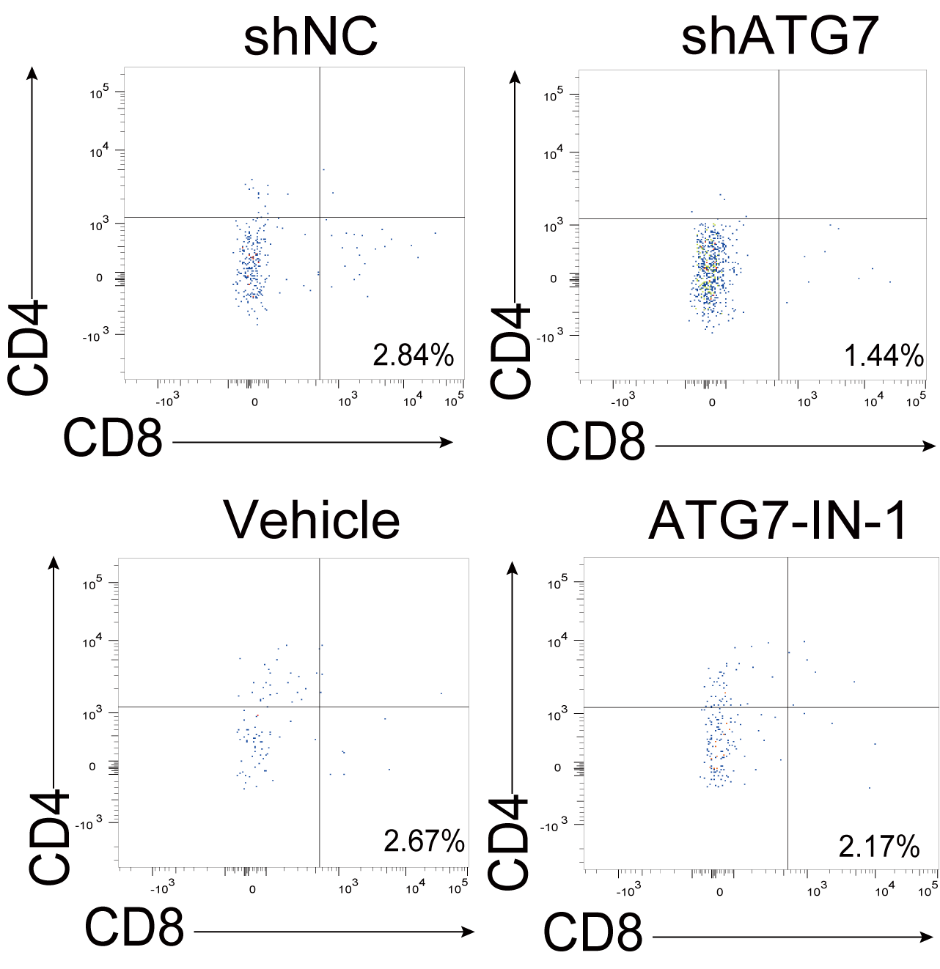


**Supplementary Fig. 4.**


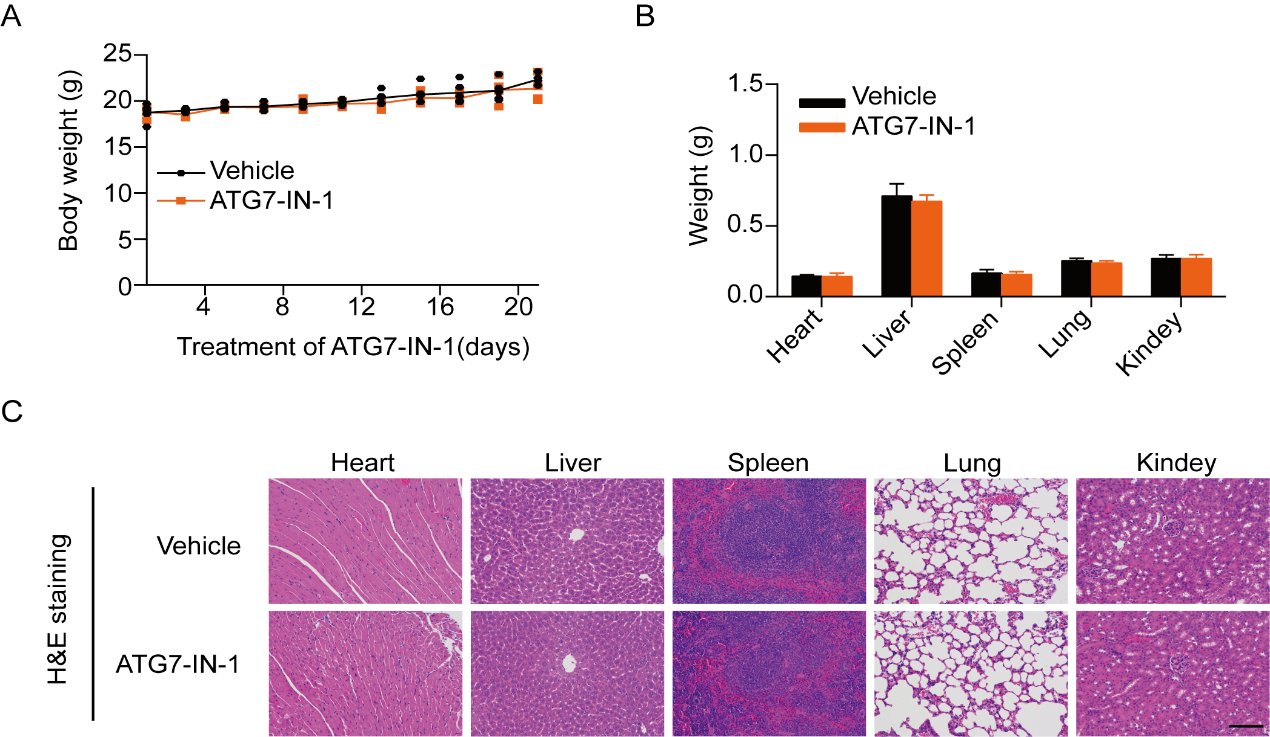


**Supplementary Fig.5.**


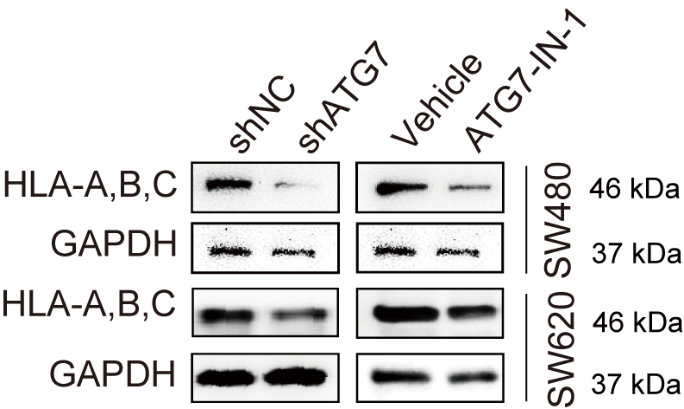


**Supplementary Fig. 6.**


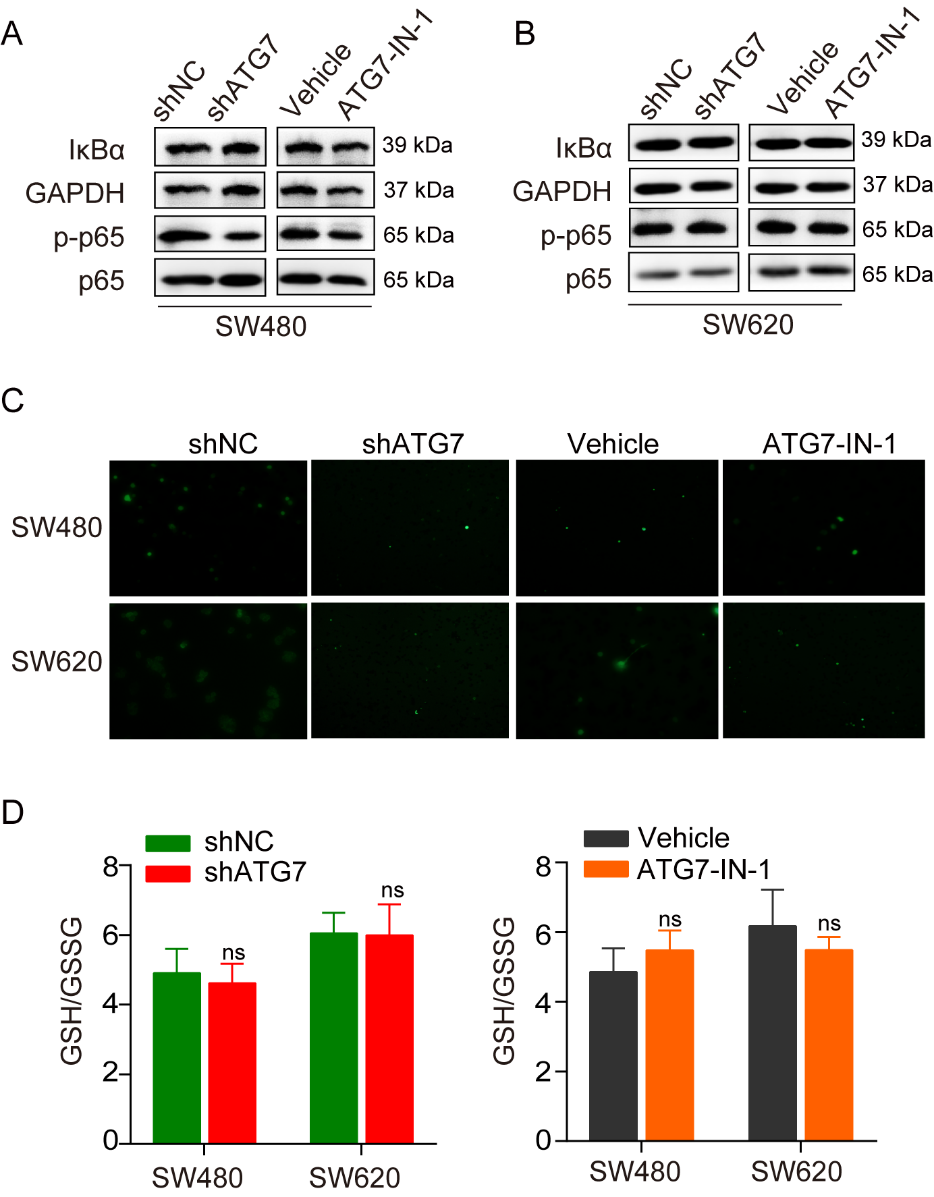


**Supplementary Table S1.**


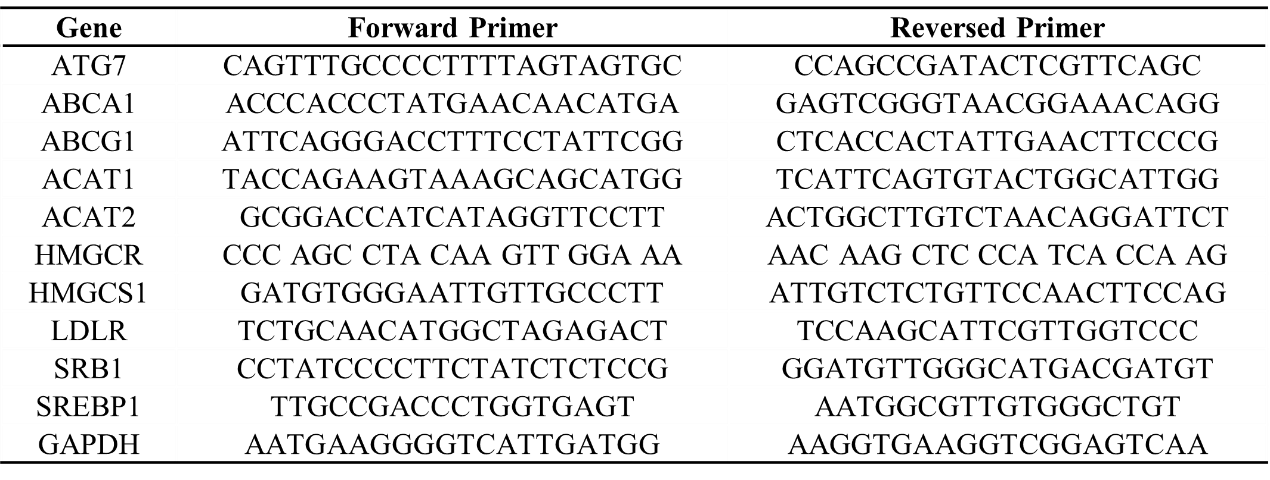


**Supplementary Table S2.**
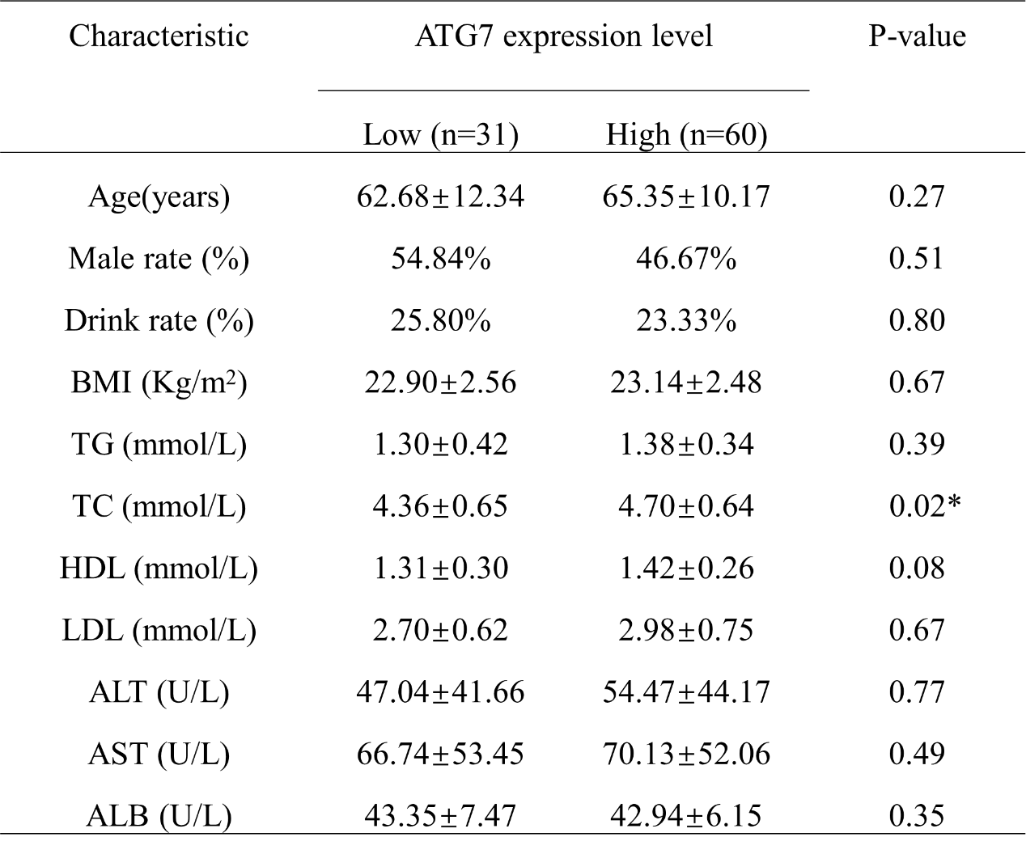

Supplement: Supplementary file 1 — Supplementary Material 1 [file 13046_2024_3023_MOESM1_ESM.docx]
